# Metabolic Effects of High Glycaemic Index Diets: A Systematic Review and Meta-Analysis of Feeding Studies in Mice and Rats

**DOI:** 10.3390/nu9070646

**Published:** 2017-06-22

**Authors:** Grace J. Campbell, Alistair M. Senior, Kim S. Bell-Anderson

**Affiliations:** 1 Charles Perkins Centre, School of Life and Environmental Sciences, University of Sydney, Sydney, NSW 2006, Australia; kim.bell-anderson@sydney.edu.au; 2 Charles Perkins Centre, School of Mathematics and Statistics, University of Sydney, Sydney, NSW 2006, Australia; alistair.senior@sydney.edu.au

**Keywords:** glycaemic index, glycaemic index, mice, rats, metabolism, glucose homeostasis

## Abstract

Low glycaemic index (LGI) diets are often reported to benefit metabolic health, but the mechanism(s) responsible are not clear. This review aimed to systematically identify studies investigating metabolic effects of high glycaemic index (HGI) versus LGI diets in mice and rats. A meta-analysis was conducted to calculate an overall effect size, Hedge’s standardised mean differences (hereafter *d)*, for each trait, with moderator variables considered in subsequent meta-regressions. Across 30 articles, a HGI diet increased five of the seven traits examined: body weight (*d* = 0.55; 95% confidence interval: 0.31, 0.79), fat mass (*d* = 1.08; 0.67, 1.49), fasting circulating insulin levels (*d* = 0.40; 0.09, 0.71), and glucose (*d* = 0.80; 0.35, 1.25) and insulin (*d* = 1.14; 0.50, 1.77) area under the curve during a glucose tolerance test. However, there was substantial heterogeneity among the effects for all traits and the small number of studies enabled only limited investigation of possible confounding factors. HGI diets favour body weight gain, increased adiposity and detrimentally affect parameters of glucose homeostasis in mice and rats, but these effects may not be a direct result of GI per se; rather they may be due to variation in other dietary constituents, such as dietary fibre, a factor which is known to reduce the GI of food and promote health via GI-independent mechanisms.

## 1. Introduction

Obesity is a chronic lifestyle disease that is brought about by many factors, the easiest of which to identify are diet and behaviour. Increased consumption of “Western” diets combined with increased sedentary behaviours are thought to be major contributors to the rapid rise in the prevalence of obesity worldwide [1]. Obesity is defined as an excess accumulation of fat mass and is associated with insulin resistance, increasing risk for type 2 diabetes mellitus, cardiovascular disease and some cancers. Diet is key to addressing this urgent public health issue, and the delineation of mechanisms linking dietary patterns and metabolic health is needed to establish optimal nutrition for individuals and populations.

Dietary guidelines recommend that carbohydrate, including sugars, starch and fibre, contribute roughly half the calories of our daily energy intake [2,3]. The nutritional importance of the subtypes of carbohydrate and their distinct physiological effects on health are not fully known. However, it is generally recommended that intake of added sugars be limited and wholegrain and fibre content increased [4,5]. Changes in food-processing technology since the Industrial Revolution have resulted in an increased supply and consumption of refined starchy foods, which feature heavily in a “Western” diet and are also associated with increased energy density, reduced fibre density and micronutrient dilution.

Starch can be further categorised as rapidly digestible starch (RDS), slowly digestible starch (SDS), or resistant starch (RS) based on the rate of digestion in the body. The two types of digestible starch (DS) are often distinguished based on their effect on postprandial blood glucose levels, with RDS quickly increasing and then decreasing blood glucose levels, and SDS inducing a slight rise that is maintained over a longer duration [6]. RS is the portion of carbohydrate that escapes digestion in the small intestine, and passes to the large intestine where it undergoes microbial fermentation, resulting in production of short chain fatty acids. RS may also be considered a source of fibre, as opposed to a form of carbohydrate, according to relatively new definitions of fibre [7].

The glycaemic index (GI) is used to compare different carbohydrate containing foods based on their effects on postprandial blood glucose levels in humans [8,9]. High glycaemic index (HGI) carbohydrates have a dramatic effect on blood glucose, with a quick release of glucose into the blood stream and a similarly quick dispersal. Low glycaemic index (LGI) carbohydrates result in a more gradual release of glucose that is digested and absorbed over a greater period of time. Hence, HGI carbohydrates consist mainly of RDS and LGI less so. The association between LGI and health is commonly criticized because a number of other factors lower GI, such as increased fat, protein and fibre content. While increased fibre, such as RS, within a diet can lower the GI of food [10], the well-documented beneficial effect of fibre on glucose homeostasis may be independent of its effects on postprandial glycaemia, and may act via other mechanisms such as those due to interactions among diet, microbiome and host.

Perhaps due to the more gradual perturbation in blood glucose levels, LGI foods lead to better control of blood glucose levels compared to HGI foods, particularly in type 2 diabetic patients [11,12]. To better understand this mechanism, the effect of both HGI and LGI diets on blood glucose homeostasis have been studied in humans and animals. Animal studies are essential for deeper investigation of molecular mechanisms attributed to the GI, and help isolate these effects of the environment, or diet, from genetic variability. While there have been several studies investigating the effect of dietary GI on rodent metabolism, these studies report somewhat contradictory results and it is unclear if these heterogeneous results are due to differences in experimental design and methodology, such as diet, or simply sampling variance. In this review, studies in mice and rats were identified and, after subjecting them to pre-set inclusion and exclusion criteria, data relating to macronutrient composition, carbohydrate source and their effect on metabolic characteristics were extracted and effect sizes derived providing the first meta-analysis of the effect of high glycaemic index diets in mice and rats.

## 2. Methods

### 2.1. Eligibility Criteria and Literature Search

The method for this review was adapted from Ainge et al. 2011 [13] and, as such, complied with the Cochrane Guidelines, Version 5.0.1 Part 2-General Methods for Cochrane Reviews [14]. In order to minimise bias during the search, a set of specific inclusion and exclusion criteria was specified a priori (Table 1). These criteria were obtained based on the set question this review aimed to answer: “What is the effect of a high versus low glycaemic index diet on metabolic characteristics in mice and rats?” As the aim was to observe the effect of GI, carbohydrate was required to contribute the majority of the energy composition. Additionally, sufficient protein content was necessary in order for the mice and rats to remain healthy and produce their own protein [15,16,17].

Four databases, PubMed, Web of Science, Medline and Scopus, were searched for papers containing a HGI diet fed to either mice or rats (Table 2). Boolean operators, alternate phrases and spellings, truncated search terms and quotation marks were used to ensure the search completed was as comprehensive and specific as possible. This search was conducted on 3 January 2017.

Once duplicate articles had been removed, each article’s abstract was subjected to the inclusion and exclusion criteria as described above. The remaining articles were then similarly screened based on their methodology. The reference lists of all articles that fit the inclusion and exclusion criteria were obtained and these articles similarly screened. Where information was missing, it was assumed that the article did not meet the specified standards on that point. For example, if the article did not state the macronutrient composition, it was assumed that it would not meet the requirements and was excluded.

### 2.2. Data Extraction

The full texts of the final articles were examined and requisite information was extracted, including the specific animals and diets used, and any measurement that could be used to determine the effects of the experiment on body weight, body adiposity, energy intake or glucose homeostasis. Glucose homeostasis measures included were fasting blood glucose levels, fasting plasma or serum insulin levels and the area under the curves (AUCs) for glucose and insulin during glucose tolerance tests (GTTs), leading to a total of seven traits. Data were collated primarily from text and/or tables, but where unavailable was extracted from graphs using GetData Graph Digitizer software v2.26.0.20 (GetData, Kogarah, Australia). To maximize the differences between HGI and LGI fed animals, the latest time point for each trait for each study, or subgroup of animals for studies with multiple cohorts, was used. Any data lacking sample size or a measure of sample distribution, such as standard deviation or standard error of the mean, were not included.

For each of the examined traits for each group of animals on a HGI or LGI diet, the mean trait value, a measure of error such as standard error of the mean, standard deviation or 95% confidence interval (CI) and the sample size were extracted. All measures of variation were back calculated to standard deviation for calculation of the effect size. Where a range for the sample size was given, the minimum value was used to ensure that the results were not given undeserved weighting.

Data for seven moderator variables were also extracted for each group of animals: species, sex, length of diet, dietary fat content, starting age, length of fasting time, and use of anaesthesia. Three were categorical factors (species, sex and use of anaesthesia) and four were continuous variables (length of diet, dietary fat content, starting age and length of fasting time). All categorical factors were recorded as binary variables; mouse or rat, male or female, yes or no respectively. Continuous variables were recorded as an integer, with length of diet and starting age recorded in days, dietary fat content as a percentage of the dietary energy content, and length of fasting time in hours. Where a range was provided for any of the continuous factors, the midpoint was used. An age of “adult” was assumed to be 18 weeks or 126 days, and a fasting time of “overnight” was assumed to be 15 h unless other clarifying information was given. Two papers did not provide starting age and as such were not included in that particular meta-regression. The raw data are available in Table S1. All continuous variables were scaled to standard deviations and centred via z-transformation.

### 2.3. Effect Size Calculation and Meta-Analysis

For each experimental comparison within a study, we calculated Hedge’s *d* (hereafter *d*) and its sampling variance following equations 1–4 in Nakagawa et al. 2015 [18]. We calculated *d* such that positive values suggest that the trait of interest was greater in the HGI group than the LGI group of animals. All effect size calculations and meta-analyses were implemented using the statistical programming language *R* v3.3.1 (The R Foundation, Vienna, Austria) [19]. The seven traits were analysed separately, but in an identical way. To estimate the overall effect size for each trait we first implemented a random-effects meta-analysis (REMA), using the “rma.mv” function in the *R* package *metafor* (Maastricht University, Maastricht, Netherlands) [20]. Each REMA fitted *d* as the response along with its sampling variance, as well as a matrix denoting the estimated covariance among effect sizes that were based on contrasts with the same control group of animals, following the method of Besson et al. 2016 [21]. This type of covariance is sometimes called non-independence due to shared control or “stochastic dependency” [22,23]. Statistical significance of the overall effect of the diet was identified when the 95% CI did not span 0. *d* values of 0.3, 0.5 and 0.8 were considered small, moderate and large differences, respectively [18]. Excluding *p*-values, all statistical results are reported to two decimal places. We report statistical heterogeneity, that is, variation among studies in the reported effect of diet on each trait, as *τ* and assess its statistical significance using Cochran’s *Q* test [24]. *τ* is interpretable as the standard deviation of the effect sizes, that is, differences in study outcomes that is not attributable to sampling.

In a second step, we explored possible drivers of heterogeneity by fitting random-effects meta-regression (REMR) to explore factors that affected the magnitude of the reported effects. REMR were fitted in the same way as the REMA described above, but with moderator variables included. The effect of each moderator variable on each trait was evaluated in a separate model. The restriction of limited sample sizes precluded the consideration of multiple predictors simultaneously, thus each moderator was evaluated individually. The moderators chosen were those considered most crucial to the methodology, and as such were also those most likely reported. Due to limited sample sizes, only those moderators that were disclosed in most papers could be analysed.

We evaluated the impact of publication bias, which is a systematic under-representation of effect sizes owing to the process of peer-review and publishing, using a trim and fill analysis with the “trimfill” function in *metafor*. Trim and fill analyses were applied to the residuals of the REMA for each trait following the method of Nakagawa et al. 2012 [25]. All *R* code is presented in Text S1.

### 2.4. Methodological Quality Assessment (MQA)

To assess the quality and validity of the articles, we performed a MQA twice by independent researchers (G.J.C. and K.S.B.-A.). The criteria were obtained from Ainge et al. 2011 [13] and as such were a modified Downs and Black “Quality Index” (QI) (Table 3) [26]. There were two categories, each with three sections. These criteria were further modified for this review with an additional 4 criteria added to the Design and Outcomes section within the Reporting category, and one removed from the Confounding section in the Internal Validity category. There were further slight modifications to the specific questions in order to tailor it to this review.

For the quality assessment, if a specific point was not addressed in the article, such as blinding or the sample size for the power calculation, the article achieved a 0 for that question. The qualification of the final MQA question, concerning power, was determined using the software program G*Power v3.1.9.2 (Heinrich-Heine-Universität, Düsseldorf, Germany) [27]. A one tailed unpaired *t*-test post-hoc analysis was performed with the effective size of a difference of 1.0 mM for plasma glucose, α, the probability of incorrectly rejecting the null hypothesis, set to 0.05, and the accepted power threshold of above 0.8. That is, an accepted threshold of β, the probability of incorrectly retaining the null hypothesis, of below 0.2.

## 3. Results

### 3.1. Article Selection

**Figure 1:**
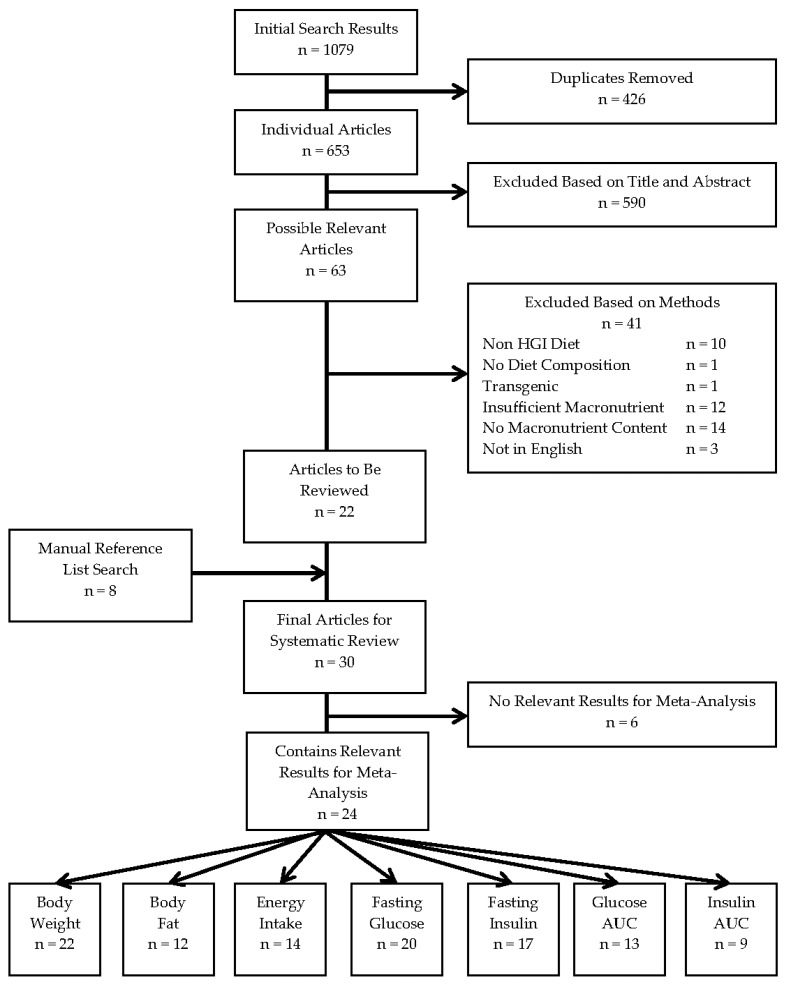
Flow of the study selection process for this systematic review. A flow diagram of article numbers included and excluded at each step of the systematic review process. Adapted from Ainge et al. 2011 [13]. *n*, number of articles; HGI, high glycaemic index; AUC, area under the curve.

Twenty-two articles were identified from the initial search, with an additional eight from the manual search of reference lists. The systematic search procedure with the number of articles included and excluded at each point is depicted in Figure 1. The end result was 30 articles, with 15 [28,29,30,31,32,33,34,35,36,37,38,39,40,41,42] involving mice, 13 [43,44,45,46,47,48,49,50,51,52,53,54,55] involving rats and two [56,57] with experiments performed on both species. Of the articles that contained pertinent results, all contained information regarding the change or comparison of body weight or fat, and most examined some measure of glucose homeostasis. Each study also included a LGI diet as a comparison for the HGI diet; only two also included a chow diet as a control [28,46] and neither reported the in vivo postprandial glycaemic response to chow, which would have led to severely underpowered analyses; thus, only LGI control diets were analysed. No study used the standard rodent diets from the American Institute of Nutrition (AIN), AIN-93, as control diets. The average study quality was moderate with a range of 8–18 and a median of 14, and a range of 10–16 with a median of 12 for mice and rats, respectively.

### 3.2. Meta-Analysis

**Figure 2:**
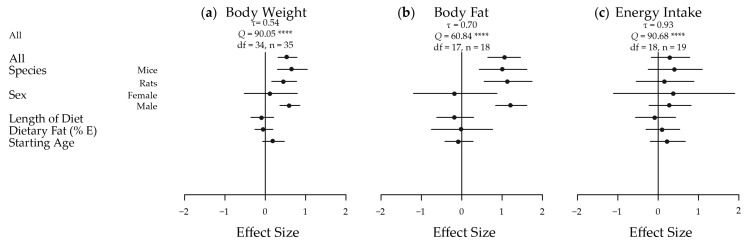
Meta-analysis of the extracted results for body weight, body fat and energy intake in high versus low glycaemic index fed animals. A plot of the calculated effect size and sampling variance for each of the three traits either as a whole or broken down by confounding factor: (**a**) Body Weight; All, Length of Diet and Dietary Fat *n* = 35, Mice *n* = 14, Rats *n* = 21, Female *n* = 4, Male *n* = 31, Starting Age *n* = 32; (**b**) Body Fat; All, Length of Diet and Dietary Fat *n* = 18, Mice *n* = 9, Rats *n* = 9, Female *n* = 2, Male *n* = 16, Starting Age *n* = 17; and (**c**) Energy Intake; All, Length of Diet and Dietary Fat *n* = 19, Mice *n* = 10, Rats *n* = 9, Female *n* = 2, Male *n* = 17, Starting Age *n* = 18. *n*, number of effect sizes; *df*, degrees of freedom; E, energy. **** *p* < 0.0001

**Figure 3:**
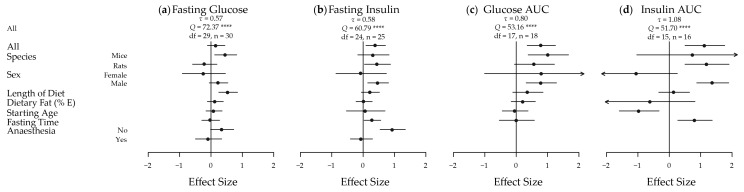
Meta-analysis of the extracted results for measures of glucose homeostasis in high versus low glycaemic index fed animals. A plot of the calculated effect size and sampling variance for each of the four traits either as a whole or broken down by confounding factor: (**a**) Fasting Glucose; All, Length of Diet, Dietary Fat and Fasting Time *n* = 30, Mice *n* = 16, Rats *n* = 14, Female *n* = 4, Male *n* = 26, Starting Age *n* = 27, No Anaesthesia *n* = 17, Anaesthesia *n* = 13; (**b**) Fasting Insulin; All, Length of Diet, Dietary Fat and Fasting Time *n* = 25, Mice *n* = 9, Rats *n* = 16, Female *n* = 3, Male *n* = 22, Starting Age, *n* = 23, No Anaesthesia *n* = 12, Anaesthesia *n* = 13; (**c**) Glucose AUC; All, Length of Diet, Dietary Fat and Fasting Time *n* = 18, Mice *n* = 9, Rats *n* = 9, Female *n* = 1, Male *n* = 17, Starting Age *n* = 17; and (**d**) Insulin AUC; All, Length of Diet, Dietary Fat and Fasting Time *n* = 16, Mice *n* = 2, Rats *n* = 14, Female *n* = 2, Male *n* = 14, Starting Age *n* = 14. AUC, area under the curve; *n*, number of effect sizes; *df*, degrees of freedom; E, energy. **** *p* < 0.0001

The meta-analyses of the extracted results, in terms of body weight, body adiposity and energy intake, and the results in relation to key measures of glucose homeostasis, are depicted in Figure 2 and Figure 3, respectively. These results are shown dissected in terms of species, sex, length of diet, dietary fat content, starting age, fasting time and use of anaesthesia to illustrate some of the factors influencing results. Five of the seven traits showed an increased effect in male animals fed a HGI diet. Fasting blood glucose was only significantly increased by HGI feeding in male mice, and no significant effects were detected in energy intake. A funnel plot of the effect sizes for each trait is shown in Figure S1.

#### 3.2.1. Body Weight

Overall, there was a moderate and statistically significant increase in body weight in HGI fed animals relative to LGI fed animals (*d* = 0.55; 95% CI: 0.31, 0.79) (Figure 2a). Significant heterogeneity was present across the studies (*τ* = 0.54, *Q* = 90.05, degrees of freedom (*df*) = 34, *p* < 0.0001) necessitating further analysis. When subset, this effect was significant and large in males (*d* = 0.61; 0.36, 0.86) but not in females (*d* = 0.13; −0.52, 0.47). The overall effect was also slightly greater in mice (*d* = 0.67; 0.30, 1.04) than in rats (*d* = 0.47; 0.16, 0.78). Starting age, length of diet and percentage of dietary fat as energy were not estimated to have any effect on the magnitude of the effect of diet on body weight. The effect on body weight was most prominent in ad libitum studies, and as most of the studies were indeed ad libitum, the results reflected this. For example, rats fed ad libitum HGI diets exhibited a much greater body weight than those fed a LGI diet in many of the studies [43,45,47,50,51,57], but, in contrast, rats fed energy-restricted HGI diets lost a greater proportion of body weight than those fed an energy-restricted LGI diet [50].

#### 3.2.2. Body Fat Mass

Body fat mass also had a large and significant increase in HGI compared to LGI fed animals overall (*d* = 1.08; 0.67, 1.49) (Figure 2b), again there was significant heterogeneity amongst effects (*τ* = 0.70, *Q* = 60.84, *df* = 17, *p* < 0.0001). This effect was also large and significant in mice (*d* = 1.02; 0.43, 1.62), rats (*d* = 1.15; 0.56, 1.75) and males (*d* = 1.23, 0.84, 1.62). Again, there were no significant differences in females and no moderation of the effect was observed with continuous variables. In specific studies, a HGI diet induced significantly greater body fat mass [28,29,30,31,32,33,35,36,37,42] by as much as 93% [56] compared with mice on a LGI diet. Specifically, visceral and subcutaneous fat depots were heavier [30,31], and epididymal adipocytes were larger [56], in HGI fed mice.

#### 3.2.3. Energy Intake

Energy intake was not significantly different in any subset (overall *d* = 0.31; −0.17, 0.78) (Figure 2c), and again no moderation was seen with the continuous variables. Significant heterogeneity existed amongst the effects (*τ* = 0.93, *Q* = 90.68, *df* = 18, *p* < 0.0001). Interestingly, a LGI diet increased energy output in faeces as measured by bomb calorimetry [29,32,42], compared to a HGI diet in the three papers that measured energy excretion to help inform energy balance.

#### 3.2.4. Fasting Blood Glucose Levels

While fasting blood glucose levels were not significantly different overall (*d* = 0.17; −0.11, 0.45) (Figure 3a), significant heterogeneity was present (*τ* = 0.57, *Q* = 72.37, *df* = 29, *p* < 0.0001). Mice had a moderate significant high fasting blood glucose (*d* = 0.47; 0.12, 0.82) in response to HGI feeding, and a statistically significant moderately positive effect of length of time on the diet was also seen (*d* = 0.55; 0.25, 0.85). This suggests that the longer the animals were on the diet, the greater the difference in mean fasting blood glucose levels between the HGI and LGI fed animals. 

#### 3.2.5. Fasting Circulating Insulin Levels

Fasting insulin was moderately and significantly affected overall (*d* = 0.40; 0.09, 0.71) (Figure 3b), with significant heterogeneity (*τ* = 0.58, *Q* = 60.79, *df* = 24, *p* < 0.0001). Similar magnitude effects were seen in rats (*d* = 0.46; 0.04, 0.87) and males (*d* = 0.48; 0.14, 0.81). A large effect was seen in the subset of animals that were not anesthetized (*d* = 0.94; 0.54, 1.35). The difference in fasting insulin levels between diets also became greater with fasting time (*d* = 0.30; 0.03, 0.56). 

#### 3.2.6. Glucose and Insulin AUC

Glucose AUC in response to a GTT was significantly increased in HGI fed animals with a large difference seen overall (*d* = 0.80; 0.35, 1.25) (Figure 3c). Again, significant heterogeneity was present (*τ* = 0.80, *Q* = 53.17, *df* = 17, *p* < 0.0001). Large effects were observed in mice (*d* = 1.02; 0.38, 1.67) and in males (*d* = 0.80; 0.31, 1.29). Insulin AUC in response to a GTT was significantly and largely increased by a HGI diet overall (*d* = 1.14; 0.50, 1.77) (Figure 3d), in rats (*d* = 1.20; 0.50, 1.90) and in males (*d* = 1.39; 0.87, 1.90). Similar to fasting insulin, a large statistically significant effect was observed for fasting time on insulin AUC (*d* = 0.82; 0.27, 1.37), with significant heterogeneity (*τ* = 1.08, *Q* = 51.70, *df* = 15, *p* < 0.0001). A large negative significant effect was also seen for starting age on insulin AUC (*d* = −0.96; −1.60, −0.32). This implies that the older the animals are when they are placed on the experimental GI diets, the smaller the difference is between insulin AUC during a GTT between HGI and LGI diets, suggesting early exposure has a significantly more dramatic effect on insulin AUC.

#### 3.2.7. Publication Bias

Trim and fill analysis detected no missing studies for four of the examined traits: body weight, energy intake, fasting glucose and glucose AUC. For both body fat and fasting insulin, it was calculated that there were four studies missing to the left side of the funnel (Figure S1b,e). After adjustment for these missing studies, the overall effects for body fat and fasting insulin where decreased by 0.25 to 0.84, and by 0.27 to 0.13, respectively. In the case of fasting insulin, this adjusted mean effect constitutes a small effect, as opposed to a moderate effect. Insulin AUC was estimated to have one missing study from the left of the funnel (Figure S1g), which reduced the effect size by 0.07 and led to an adjusted overall effect of 1.06, not altering the qualitative interpretation of the magnitude of the effect, similar to body fat.

### 3.3. MQA

The animal and diet characteristics for each of the studies are shown in Table 4 and Table 5 for mice and rats, respectively, listed by year. The results for the MQA are shown in Table 6 and Table 7 for mice and rats, respectively. The average score, out of 22, for each species was 13.3 and 12.2, respectively, to one decimal place. There was a small amount of variation in the quality of the articles, with the vast majority having a score between 11 and 16, inclusive [28,29,30,32,34,35,36,37,41,42,43,44,45,47,49,50,51,52,54,55,56,57], and a lowest score of 8 [38] and highest of 18 [31].

## 4. Discussion

This systematic review and meta-analysis provides evidence that male mice and rats fed HGI diets increase body weight, body adiposity, and parameters of glucose homeostasis compared to animals fed LGI diets. These findings highlight the importance of determining the effect of different carbohydrate subtypes on metabolic health, as while slowing carbohydrate bioavailability might be key, the LGI diets were also typically high in fibre such as RS which may exert mechanisms on metabolism independent of effects on postprandial glycaemia. Unfortunately, we were not able to analyse the effect of dietary fibre on metabolic outcomes across the studies in our meta-analysis as fibre content was typically not quantified in the original reporting articles, with only two paper specifying fibre content [34,57]. This lack of information was taken into consideration when determining the quality of the papers through question 15 of the MQA. This study, as a systematic review and meta-analysis, has examined the current state of rodent GI studies in an unbiased way, and has shown that, overall, better controlled studies are required in the future to properly elucidate the effect of GI alone on metabolism.

The current literature has been shown to be insufficient in regards to examining the specific effect of GI on metabolism. Only seven traits could be examined, and only seven moderators included in analyses due to the limit reported data. To examine any other effects or correlations, significant improvement in current reporting standards is required, as any other analyses conducted on the current literature would be severely underpowered. This reviews’ calculated correlations are important new findings that can be used to plan future studies.

Animal studies are key to dissecting the mechanisms underlying the health benefits of a LGI diet, but it seems isolating the effect of postprandial glycaemia while controlling for other physiological properties of LGI foods is not widespread. The glycaemic potential of most foods is indicated by starch digestibility and glucose absorption from the gut. A number of things could affect the GI including: type of starch, fibre viscosity, the food matrix, cooking, processing and macronutrient composition. The majority of articles in this review based their GI diets on the percentages of amylose or amylopectin in the starch. It has been reported that the commonly used low GI Hi-Maize amylose starch contains up to 60% RS [59]. RS reduces the available energy density of the diet and may have important effects on gut hormone secretion as it modifies the motility of the gastrointestinal tract and interacts with the gut microbiome. RS represents energy that is consumed but not available to the host, and would explain reports of increased energy content in faecal output in LGI fed animals [29,32,42]. Several articles also used RS and a control of a HGI starch. However, during the search for articles, we did not include a search term specific to RS as this review aimed to isolate articles that tested diets with differing GI, rather than RS content. This was regardless of the fact that many articles seemed to view these two concepts as synonymous. However, based on the premise for a systematic review, any articles that met the criteria were included in this review regardless of the RS content of their diets. It is important to note that most of the LGI diets contained RS at levels that could not be achieved in human diets, which may be a differentiator of human and animal studies.

There is no standard for GI testing in animals. As shown in the MQA, only eight mouse and five rat papers showed some difference in the GI of the diets, with most others merely assuming a difference in GI based on differing levels of RS as discussed above, or the GI of the carbohydrate sources as measured in humans. Some studies contained no basis for the high versus low GI diets, other than claiming them to be so. Of the studies that did discuss a proven difference in GI, most conducted a meal tolerance test that has similarities to the standard GI practice in humans, however they were key differences in methodology that showed no study accurately measured GI in vivo. In humans, ten subjects are tested in a cross-over design where they consume the tested meal over 12 min, have their blood glucose tested over the following 2 h, and the AUC compared to an identical test with glucose matched for the amount of carbohydrate in the meal by weight [60]. Three studies examined GI in vitro [34,45,54], which has been shown to be similar to GI measured in vivo in humans [6]. Of these three, one [34] used a reference food of white bread to calculate an approximate GI, another [45] also conducted a meal tolerance test, and the last [54] performed an additional in vivo GI test in rats, but did not state the methodology. Of the eleven papers that performed meal tolerance tests, two [31,50] performed the tests in a cross-over design as would be done in human. Three papers [33,37,50], with two of these from the same study, used a reference food, although one study used glucose in the diet frame as opposed to straight glucose and while the other did test straight glucose, it was compared to the carbohydrate source as opposed to the diet itself. Three provided the diets in terms of carbohydrate content [43,50,55], as is done in humans, as opposed to food weight. Only three papers used at least 10 animals for the test [33,37,45], and the time to eat ranged considerably with two as gavages [55], three eating for 15 min [33,37,45], and the remaining tests having the animals eat for 5 min [32,35,42,43]. Most studies did test the blood glucose over two hours [32,33,35,37,43,50,54,55]. While the meal tolerance tests were able to show a qualitative difference in glycaemic response, without following the standard methodology, particularly in terms of using a glucose reference, the GI could not be quantified for any diet. Regrettably no study examined the GI of standard diets, however, recently in our laboratory we successfully implemented GI testing in mice and found that both chow and high corn starch diets, similar to AIN-93, are actually relatively HGI [61].

Unfortunately, many articles lacked specific details relating to diet composition. We included six articles where the macronutrient content was stated, but not explicated to whether this percentage was of weight or energy [29,30,31,35,38,40]. Criteria for this review required carbohydrate contribution to energy greater than 50%, and, as protein, fat and carbohydrates have different energy densities, if the given content was of weight, some articles may not have actually met these criteria; accordingly, these articles were excluded. A further article [43] did not state the protein content at all, and it was only through being cited in another paper [44] that this information could be found and the original article [43] included. Future studies should report animal diet ingredients for confirmation that the diets meet all nutritional needs of the animals and enable reproducibility of experimental results.

Only one [46] of the HGI diets was paired with a control that was matched for both digestible macronutrient composition and fibre, including resistant starch, content, but this study had several limitations. Of all studies included in the meta-analysis, it was one of two studies that did not state the starting age of animals, one of only five using females, and was the shortest study overall, with rats exposed to the diet for only two weeks [46]. Of note, the rats from this paper were subjected to electrode induced lesions on the ventromedial nucleus of their hypothalamus, which would severely impact their metabolism [46]. Additionally, as this is a highly invasive surgery, even the sham rats would be significantly and adversely affected.

In the meta-regression, there were considerable differences between the sexes. Of the 30 articles included, five papers detailed experiments on female animals, one of which used mice [34] and four used rats [46,54,55,57]. This makes it difficult to be sure that there is really a sex difference in response to HGI feeding as the data suggest. Traditionally, female animals, as opposed to male, have been thought to present additional variation due to the continually changing hormonal environment through the oestrous cycle. One of the papers that used female rats also used male rats in a parallel experiment, however the sample size for female rats was five times smaller leading to insufficient power [57]. Ideally, equivalent studies should be performed, and results compared, in both sexes of mice and rats as it is becoming increasingly apparent that there are distinct metabolic differences between the sexes [62,63] necessitating the investigation of both sexes in order to gain full understanding of any metabolic factors. The dearth of female studies is most likely a crucial contributor to the lack of significant results for this subgroup for any of the traits. 

Several articles did not use conventional animal strains. A “conventional” animal strain refers to those used in the majority of papers, such as C57BL/6 mice, and Albino Wistar or Sprague-Dawley rats, being the most well studied and hence understood. C57BL/6 mice are commonly used in metabolic studies, as they are susceptible to diet-induced obesity and hyperglycaemia. The animals studied within this systematic review that were not deemed “typical” include CBA/T6 [28] and 129S2/SvPas [33] mice, and spontaneously hypertensive [49], and partial pancreatomised [56] rats. The issue with the last is obvious, as any hormones the pancreas produces, not limited to insulin, would be decreased and therefore would alter the entire systemic environment. The experiments from that article [56] involving non-pancreatomised rats were still included in this meta-analysis, as were the experiments involving streptozotocin [45,57], gold thioglucose [28] or leptin [47] injections or surgery to induce lesions on the ventromedial nucleus of the hypothalamus [46] as a sensitivity test showed removing these data did not affect the analytical outcomes. A few studies [43,44,47,48] also involved inserting cannulas into the animals’ jugular vein and/or carotid artery but as this is a relatively short surgery and the animals were given sufficient recovery time, the impact on metabolic results should be minimal. All of these animal strains and treatments have the potential to confound any and all results; regardless, results reported in these animals were generally consistent with those reported across most studies in this review. For this reason, as well as the limited number of total studies, it was decided not to investigate the effects broken down by the many different species during the meta-analysis.

The animal age at commencement of study and the length of study varied considerably. Most of the mice studies [32,33,34,35,36,37,38,39,40,41,42,56,57] utilized adult animals, ranging in age from 8 weeks to 16 months at the start of the study, while several rat studies used animals that were 6 weeks old or younger [43,44,45,47,48,49,55,56]. Two rat studies did not provide the starting age of animals, thus their developmental state is unknown [46,54]. One of these studies [54] is a study of the maternal effect of diet, so it could be assumed that the rats were mature, but the other [46] gave no age indication, and only lasted for two weeks, thus these rats may not be fully developed even at the completion of the study. Most of the mice studies were relatively long studies, consisting of interventions lasting at least 16 weeks [29,31,32,34,35,36,38,39,40,41,42] compared to the majority of rat studies lasting only 4–8 weeks, with only four lasting longer than eight weeks [43,44,54,56]. Due to the young age and short length of study, it is possible that the animals in some of these experiments [30,43,45,46,47,48,49,52,53,55,56] are still in an adolescence period and hence the results may not reflect the generalized effects of HGI or LGI diets. Indeed, the results of the meta-analysis show that early and longer exposure significantly increase the effect of HGI diets on insulin AUC and fasting glucose, respectively. This should be taken into account when planning a GI animal experiment.

The MQA question on power relied on sample size, hence the eight studies that did not provide sample size [38,40,42,43,46,47,48,49,57] received a potentially lower MQA score for this lack of information. Of all 30 articles, only eight had sufficient power [30,31,33,37,44,45,54,55] with a sample size of 14 or greater per group. Most of the studies had a sample size of 8–12, with the smallest sample size of six [36] and highest of 50 [33,37,55,57]. The insufficient replication in the papers with smaller sample sizes is indicative of less reliable results which require further investigation before a valid conclusion can be made and accepted. However, as the calculation for the effect size, and the associated estimate of sampling variance which was included in the analyses, was dependant on the sample size, our analyses accounted for differences among studies arising due to precision. Thus, all studies were included in the meta-analysis to avoid this bias.

Two questions we added to the MQA were regarding use of anaesthetic and fasting time. Most studies refrained from using anaesthesia, with only three studies using anaesthetic during non-terminal blood sampling [34,45,51]. No study used anaesthesia for GTTs. Anaesthesia affects brain metabolism and can affect serum concentrations of circulating proteins and metabolites [64], thus use of anaesthesia for metabolic studies is not desirable as it may impair the accurate measurement of postprandial changes in glucose and metabolic hormones such as insulin. The time frame used for acceptable fasting was 5 h. Excessive fasting, such as overnight for mice or greater than 18 h for rats, can artificially induce larger differences in the data [64]. Increased fasting time was shown to increase the effect of HGI on fasting insulin or insulin AUC to a small or large extent respectively. Fasting insulin was also affected by anaesthesia, with an absence of anaesthesia correlating with a large difference between HGI and LGI fed animals, and those subjected to anaesthesia showing no significant difference between the diets. Thus, use of anaesthesia may mask effects and fasting time may exaggerate effects on glucose metabolism and are important considerations in metabolic studies.

Most of the articles included measured some parameter of glucose homeostasis. The majority measured only blood glucose and insulin levels, with a few performing GTTs of some form. Only one paper conducted a euglycaemic clamp [48]; however, the findings are questionable given that the insulin levels reported were too high for animal survival, with a peak at 4200 pmol/L [48] compared with a typical peak being an average of 1200 pmol/L [65,66,67]. Additionally, the glucose metabolism calculations in this paper were not physiological [48]. All results in this paper therefore, must be seen as questionable. 

The two articles by Van Schothorst et al. [33,37] contained one study, split over two papers. While the results were not duplicated as such, the continuation of a single study across the two articles is worth noting since it leads to a replication of similar findings included in this review from the two papers. The only relevant trait discussed in the latter paper [37], body weight, was already reported for the larger group of mice in the first article [33] and so was not included in the meta-analysis to avoid duplication.

## 5. Conclusions

This is the first systematic review and meta-analysis of the effects of GI on rodent metabolism. A HGI diet fed to male mice and rats increases body weight, adiposity, fasting insulin levels, and the AUCs for glucose and insulin levels during a GTT, to a greater extent than a LGI diet. There are too few studies in female animals to be confident of effects; future experiments should at least include females, if not specifically investigate the effects of GI diets in females given that the maternal nutritional environment is critical for the development of chronic diseases later in life. From these articles, it is difficult to conclude whether the beneficial health effects of a LGI diet are due to alterations in postprandial glycaemia per se, as dietary composition was considerably variable, particularly with respect to digestible macronutrient composition, fibre and resistant starch.

## Figures and Tables

**Table 1:** Inclusion and exclusion criteria. This criterion was used to assess all articles and determine their eligibility for inclusion in this review. HGI, high glycaemic index; LGI, low glycaemic index.

Inclusion Criteria	Exclusion Criteria
Mice or rat studies	Transgenic animal models
Experiments performed in live whole animals	Cell cultures
HGI diet	No control diet (controls include chow or LGI)
Carbohydrates >50% of energy content	Diet of unknown macronutrient composition
Proteins >19% of energy content	Use of HGI supplement such as sugars to non-HGI diet
In English	Review, conference abstract or supplementary stub article

**Table 2:** Search criteria and initial results. These specific terms were used to search four databases, with the number of results yielded stated. HGI, high glycaemic index; GI, glycaemic index.

**Search Term**	(“high glycemic*” OR “high glycaemic*” OR “high-glycemic*” OR “high-glycaemic*” OR “HGI” OR “H-GI” OR “H GI” OR “high GI” OR “high-GI” OR “simple carbohydrate*” OR “simple-carbohydrate*” OR “high carbohydrate*” OR “high-carbohydrate*” OR “high glucose*” OR “high-glucose*” OR “higher GI*” OR “higher-GI*” OR “higher glycemic*” OR “higher-glycemic*” OR “higher glycaemic*” OR “higher-glycaemic*”) AND (mice OR rat) AND (“GI” OR “glycemic*” OR “glycaemic*”)				
**Database**	PubMed	Web of Science	Scopus	Medline	Total
**Results**	175	422	228	254	1079

**Table 3:** Methodological Quality Assessment questions. MQA questions modified from Ainge et al. 2011 [13] and Downs and Black 1998 [26]. GTT, glucose tolerance test; GI, glycaemic index.

Modified Downs and Black Quality Index
*Reporting*
General
1. Were the hypotheses, aims or objectives of the study clearly described in the introduction? ^a^
2. Were the main outcomes to be measured clearly described in the introduction or methods section?
Animal Characteristics
3. Was animal species/strain and sex specified? ^a^
4. Was the animal age at commencement of the study specified? ^a^
5. Have the animal weights at commencement of the study been specified or given graphically? ^a^
6. Have the animal starting numbers been specified? ^a^
7. Have the housing details been specified, including temperature, light cycle and group housing?
Design and Outcomes
8. Were the interventions of interest clearly described?
9. Were the main findings of the study clearly described?
10. Were estimates of the random variability in the data for the main outcomes clearly described, such as through standard deviation or standard error of the mean? ^a^
11. Have all important adverse events that may be a consequence of the intervention been reported?
12. Have the actual probability values been reported for the main outcomes, except where probability value is less than 0.05? ^a^
13. Were all blood tests, GTTs performed without anaesthetic? ^b^
14. Were all GTTs performed after a maximum 5 h fast? ^b^
15. Were the diet matched both in terms of macronutrient composition and fibre content? ^b^
16. Was a difference in GI of the two diets shown, either quantitatively or qualitatively? ^b^
*Internal Validity*
Bias
17. Was an attempt made to blind those measuring the main outcomes of the intervention?
18. Were the statistical tests used to assess the main outcomes appropriate?
19. Were the main outcomes measures used accurate (valid and reliable)?
Confounding
20. Was it stated in the text that the animals were randomised to intervention groups? ^a^
21. Was there adequate adjustment for confounding in the analyses from which the main findings were drawn?
Power
22. Was the paper of sufficient power to detect a clinical important effect where the probability value for a difference being due to chance is less than 5%?

^a^ Modified questions; ^b^ Additional questions.

**Table 4:** Mouse animal and diet characteristics for intervention studies that compared LGI and HGI diets. The key information from each article using mice is shown. - denotes missing data. Approximate (~) starting weights are taken from graphs ^a^. Carb, carbohydrates; HGI, high glycaemic index; LGI, low glycaemic index; E, energy; *n*, sample size; AP, amylopectin; AL, amylose; M, male; DS, digestible starch; RS, resistant starch; F, female.

Article	Carb in HGI Diet	Carb in LGI Diet	*n*	Starting Age	Sex	Strain	Starting Weight (g)	Length of Diet	Carb (% E)	Protein (% E)
Walker, 2002 [28]	Mazaca wax 100% AP	Hi-Maize 60% AL 40% AP	12	6 weeks	M	CBA/T6	24.6 ± 0.2	10 weeks	67	22
Pawlak, 2004 [56]	100% AP	60% AL 40% AP Hi-Maize	12	11 weeks	M	C57BL/6J	26.98 ± 0.37	9 weeks	69	20
Scribner, 2007 [29]	100% AP	60% AL 40% AP	9	5 weeks	M	129S2/SvPas	~17.5	25 weeks	68	19
So, 2007 [30]	DS Amioca 0% RS	RS Hi-Maize 60% RS	24 HGI or 16 LGI	3 weeks	M	C57BL/6	~17	8 weeks	69	20
Scribner, 2008 [31]	100% AP	60% AL 40% AP	16	5 weeks	M	129SvPas	~20	10 or 38 weeks	68	19
Zhou, 2008 [57]	Amioca 100% corn AP	Hi-Maize 260	-	Adult	M	C57BL/6J	-	19 days	64 ^a^	19.3 ^a^
Isken, 2009 [32]	100% AP	30% AP	10 or 8	16 or 44 weeks	M	C57BL/6	~23	20 or 26 weeks	65	23
Van Schothorst, 2009 [33]	100% AP	~60% AL 40% AP	50	9 weeks	M	C57BL/6JOlaHsol	25.68 ± 0.23 HGI or 25.63 ± 0.2 LGI	13–14 weeks	50	20
Anderson, 2010 [34]	Pregelatinized starch	Native starch	7	6–8 weeks	F	C57BL/6J	18.5 ± 0.2	22 weeks	68 HGI or 65 LGI	18 HGI or 22 LGI
Colbert Coate, 2010 [36]	Waxy maize 100% AP	high-AL-res 40% AP	6–8	6–8 weeks	M	C57BL/6	~22	16 weeks	65	20
Isken, 2010 [35]	100% AP	30% AP	10	16 weeks	M	C57BL/6J	~25	6 or 20 weeks	65	23
Van Schothorst, 2011 [37]	100% AP	~60% AL and ~40% AP	50	9 weeks	M	C57BL/6	25.7 ± 0.2 HGI or 25.6 ± 0.2 LGI	13–14 weeks	50	20
Uchiki, 2012 [38]	100% AP	30% AP 70% AL	-	16 months	-	C57BL/6	-	7.5 months	65	21
Weikel, 2012 [39]	100% AP (Amioca)	70% AL 30% AP (Hylon VII)	10	5 or 16 months	M	C57BL/6	-	26 or 46 weeks	65	21
Birarda, 2013 [40]	100% AP (Amioca)	70% AL 30% AP (Hylon VII)	-	5 months	M	C57BL/6	-	12 months	65	21
Rowan, 2014 [41]	100% AP (Amioca)	70% AL 30% AP (Hylon VII)	9–12	11 weeks	M	C57BL/6	~26	33 weeks	65	21
Kleckner, 2015 [42]	Amioca waxy 100% AP	H-Maize 260 60% AL	-	6–8 weeks	M	C57BL/6	~26	16 weeks	66.5	20.1

^a^ Based on Ain-93G [58].

**Table 5:** Rat animal and diet characteristics for intervention studies that compared LGI and HGI diets. The key information from each article using rats is shown. - denotes missing data. Approximate (~) starting weights are taken from graphs. Articles using two different strains or sexes are across two rows, with the defining characteristics in bold. Only new information is entered in the second row. Carb, carbohydrates; HGI, high glycaemic index; LGI, low glycaemic index; E, energy; *n*, sample size; SHR, spontaneously hypertensive rats; AL, amylose; M, male; SD, Sprague-Dawley; AP, amylopectin; F, female.

Article	Carb in HGI Diet	Carb in LGI Diet	*n*	Starting Age	Sex	Strain	Starting Weight (g)	Length of Diet	Carb (% E)	Protein (% E)
Byrnes, 1995 [43]	Waxy cornstarch (0% AL)	Hi-Maize 60% AL	-	8 weeks	M	SD	250–300	8 weeks	60	22
				3–4 weeks	-	Wistar	50–90	4, 8 or 12 weeks		
Higgins, 1996 [44]	100% glucose or 100% AP (waxy corn)	Hi-Maize 60% AL	18	6 weeks	M	Wistar	200	8, 16 or 52 weeks	67	22
Lerer-Metzger, 1996 [45]	French toast (wheat starch)	Mung bean starch	14	6 weeks	M	SD	155	5 weeks	59	22 HGI or 24 LGI
Suga, 2000 [46]	60% glucose	60% fructose	-	-	F	SD	240–280	2 weeks	60	29
Widdup, 2000 [47]	Glucose	Hi-Maize 60% AL	-	6 weeks	M	Wistar	180 ± 2	6 weeks	65	22
Pawlak, 2001 [48]	Waxy corn 100% AP	Hi-Maize 60% AL	-	6–7 weeks	-	Wistar	215 ± 6	7 weeks	69	20
Pawlak, 2004 [56]	100% AP	60% AL Hi-Maize	12	6 weeks	M	SD	50–55	18 weeks	69	20
			7	7 weeks			-	3 weeks per diet		
Kopilas, 2007 [49]	50% Sucrose	50% LGI Starch	-	6 weeks	M	SHR	-	6 weeks	67.7 ^b^	20.8 ^b^
Zhou, 2008 [57]	Amioca 100% corn AP	Hi-Maize 260	50	-	M	SD	-	10 days	64 ^a^	19.3 ^a^
			10		F			32 days		
Aziz, 2009 [50]	100% AP	70% AL cornstarch or Cornstarch	10–12	Adult	M	SD	250	4 weeks	64 ^a^	19.3 ^a^
Belobrajdic, 2012 [51]	Low AL maize	High AL maize starch	8	9 weeks	M	SD	329 ± 5	4 weeks	64 ^a^	19.3 ^a^
Ble-Castillo, 2012 [52]	67% digestible cornstarch	67% native banana starch	30 total	7 weeks	M	Wistar	180–200	8 weeks	75.6	21.8
Stavrovskaya, 2013 [53]	High (65%) sucrose	Low (0%) sucrose	8	8 weeks	M	FBNF1	188 ± 2	8 weeks	68	21
Gugusheff, 2015 [54]	Dextrinised starch	Gel crisp starch	14	-	F	Wistar	200	70weeks	63	21
Thompson, 2016 [55]	Amioca corn (high AP)	Resistant starch (high AL)	50	19 days	F	SD	~40	8 weeks	50	20

^a^ Based on Ain-93G [58]; ^b^ Based on Ain-76A [58].

**Table 6:** Mouse Methodological Quality Assessment. Each article using mice was given a score of 0 or 1 for each question. MQA, methodological quality assessment.

Article	Reporting Questions																Internal Validity Questions						Total
Author, Year	1	2	3	4	5	6	7	8	9	10	11	12	13	14	15	16	17	18	19	20	21	22	
Walker, 2002 [28]	1	1	1	1	1	1	0	0	1	1	0	1	1	0	0	0	0	1	1	0	0	0	**12**
Pawlak, 2004 [56]	1	1	1	1	1	1	0	1	0	1	1	1	1	0	0	1	0	1	1	1	1	0	**16**
Scribner, 2007 [29]	0	1	1	1	1	1	0	1	1	1	0	1	1	1	0	0	0	1	1	1	1	0	**15**
So, 2007 [30]	1	1	1	1	1	1	1	1	1	1	0	1	1	0	0	0	1	1	1	0	0	1	**16**
Scribner, 2008 [31]	1	1	1	1	1	1	0	1	1	1	0	1	1	1	0	1	0	1	1	1	1	1	**18**
Zhou, 2008 [57]	0	1	1	0	0	0	1	1	1	1	0	1	1	1	0	0	0	1	1	0	0	0	**11**
Isken, 2009 [32]	1	1	1	1	1	1	1	1	0	1	0	1	1	0	0	1	0	1	1	0	0	0	**14**
Van Schothorst, 2009 [33]	1	1	1	1	1	1	1	1	1	1	0	1	1	1	0	1	0	1	1	0	0	1	**17**
Anderson, 2010 [34]	1	1	1	1	1	1	0	1	1	1	0	0	0	0	0	1	0	1	0	1	0	0	**12**
Colbert Coate, 2010 [36]	1	1	1	1	1	1	0	1	1	1	0	1	1	0	0	0	0	1	1	0	0	0	**13**
Isken, 2010 [35]	0	1	1	1	1	1	1	1	1	1	0	1	1	0	0	1	0	1	1	0	0	0	**14**
Van Schothorst, 2011 [37]	0	1	1	1	1	1	1	1	1	1	0	1	1	1	0	1	0	1	1	0	0	1	**16**
Uchiki, 2012 [38]	0	1	0	1	0	0	0	1	1	1	0	1	1	0	0	0	0	0	0	1	0	0	**8**
Weikel, 2012 [39]	0	1	1	1	0	1	0	1	1	1	0	0	1	0	0	0	0	1	0	0	0	0	**9**
Birarda, 2013 [40]	1	1	1	1	0	0	0	1	1	1	0	1	0	0	0	0	0	1	0	0	0	0	**9**
Rowan, 2014 [41]	1	1	1	1	1	1	0	0	0	1	0	1	1	0	0	0	0	1	1	0	0	0	**11**
Kleckner, 2015 [42]	1	1	1	1	1	0	1	1	1	1	0	1	1	0	0	1	0	1	1	0	1	0	**15**
**Total**																							**Mean**
**17**	**11**	**17**	**16**	**16**	**13**	**13**	**7**	**15**	**14**	**17**	**1**	**15**	**15**	**5**	**0**	**8**	**1**	**16**	**13**	**5**	**4**	**4**	**13.3**

**Table 7:** Rat Methodological Quality Assessment; each article using rats was given a score of 0 or 1 for each question. MQA, methodological quality assessment.

Article	Reporting Questions																Internal Validity Questions						Total
Author, Year	1	2	3	4	5	6	7	8	9	10	11	12	13	14	15	16	17	18	19	20	21	22	
Byrnes, 1995 [43]	1	1	1	1	1	0	0	1	1	1	0	1	1	0	0	1	0	1	0	0	0	0	**12**
Higgins, 1996 [44]	1	1	1	1	1	1	1	1	1	1	0	1	1	0	0	0	0	1	1	1	0	1	**16**
Lerer-Metzger, 1996 [45]	1	1	1	1	1	1	0	0	1	1	0	1	0	0	0	1	0	1	0	1	0	1	**13**
Suga, 2000 [46]	1	1	1	0	1	0	0	0	0	1	0	0	0	1	1	0	0	1	1	0	1	0	**10**
Widdup, 2000 [47]	1	1	1	1	1	0	1	1	1	1	0	1	1	0	0	0	0	1	1	0	0	0	**13**
Pawlak, 2001 [48]	1	1	0	1	1	0	0	1	0	1	0	1	1	0	0	0	0	1	0	1	0	0	**10**
Pawlak, 2004 [56]	1	1	1	1	0	0	0	1	1	0	1	1	1	0	0	0	0	0	1	1	1	0	**12**
Kopilas, 2007 [49]	1	1	1	1	0	0	1	1	1	1	0	1	0	0	0	0	0	1	0	0	1	0	**11**
Zhou, 2008 [57]	0	1	1	0	0	1	1	1	0	1	0	1	1	1	0	0	0	1	1	0	0	0	**11**
Aziz, 2009 [50]	1	1	1	0	1	1	1	1	1	1	0	1	1	0	0	1	0	1	0	0	0	0	**13**
Belobrajdic, 2012 [51]	1	1	1	1	1	1	1	1	1	1	0	1	0	0	0	0	0	1	1	1	0	0	**14**
Ble-Castillo, 2012 [52]	1	1	1	1	1	0	1	1	0	1	0	1	1	0	0	0	0	1	0	0	0	0	**11**
Stavrovskaya, 2013 [53]	0	0	1	1	1	1	0	1	1	1	0	1	0	0	0	0	0	0	1	1	0	0	**10**
Gugusheff, 2015 [54]	1	1	1	0	1	1	1	1	1	1	0	1	1	0	0	1	0	1	1	0	0	1	**15**
Thompson, 2016 [55]	1	1	1	1	1	1	1	0	0	1	0	1	0	0	0	1	0	0	0	1	0	1	**12**
**Total**																							**Mean**
**15**	**13**	**14**	**14**	**11**	**12**	**8**	**9**	**12**	**10**	**14**	**1**	**14**	**9**	**2**	**1**	**5**	**0**	**12**	**8**	**7**	**3**	**4**	**12.2**

## Supplementary Materials

The following are available online at www.mdpi.com/2072-6643/9/7/646/s1, Table S1: Raw data for meta-analysis, Text S1: R code for meta-analysis, Figure S1: Funnel plot of effect sizes.

## Abbreviations

AIN	American Institute of Nutrition
AUC	area under curve
CI	confidence interval
*df*	degrees of freedom
DS	digestible starch
GI	glycaemic index
GTT	glucose tolerance test
HGI	high glycaemic index
LGI	low glycaemic index
MQA	methodological quality assessment
QI	quality index
RDS	rapidly digestible starch
REMA	random-effects meta-analysis
REMR	random-effects meta-regression
RQ	respiratory quotient
RS	resistant starch
SDS	slowly digestible starch
