# Supplementary material for: Metabolic Effects of High Glycaemic Index Diets: A Systematic Review and Meta-Analysis of Feeding Studies in Mice and Rats
Source: Nutrients. 2017 Jun 22;9(7):646. doi: 10.3390/nu9070646 (PMC5537766; doi:10.3390/nu9070646)
Supplement: Supplementary file 1 [file nutrients-09-00646-s001.pdf]

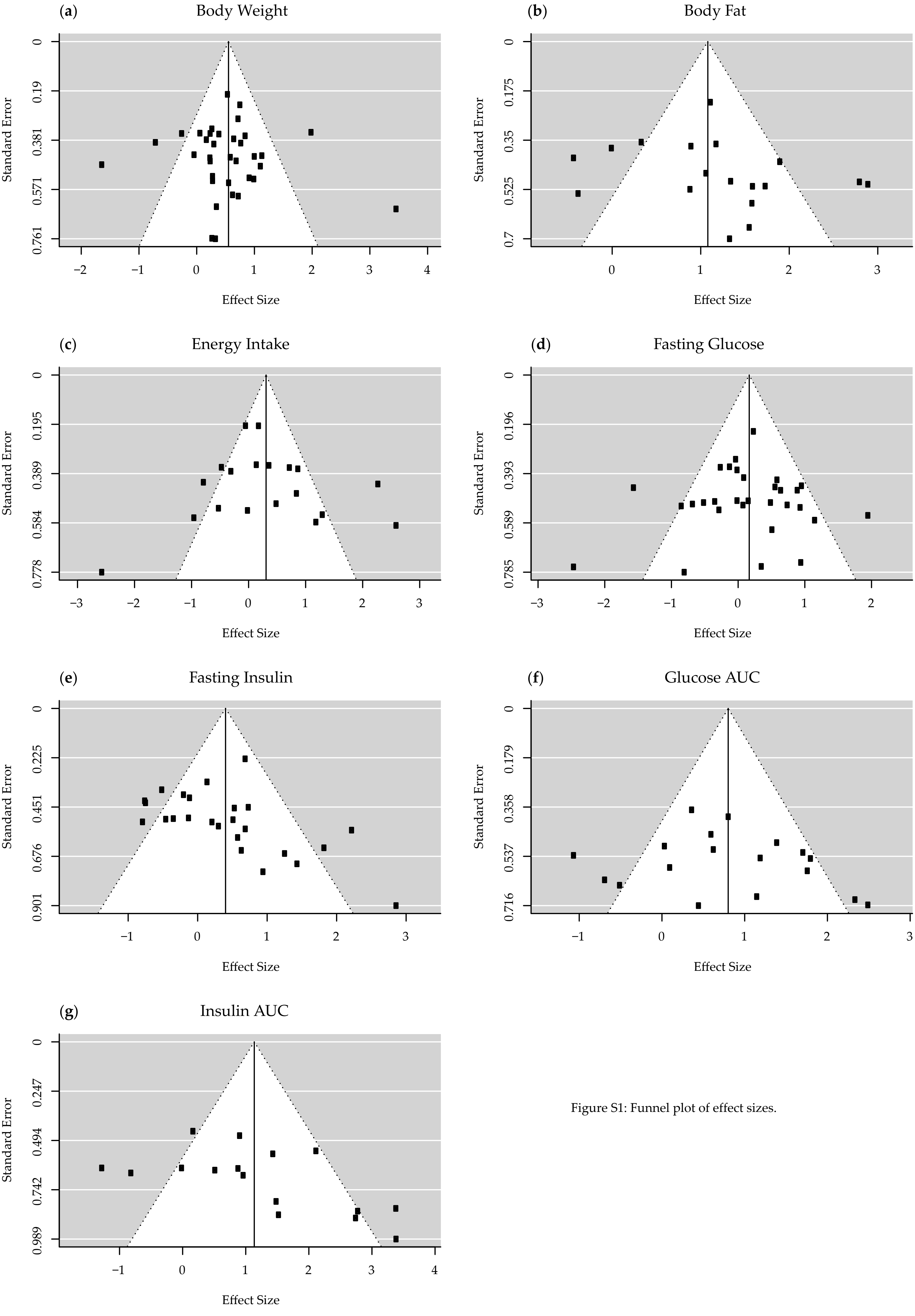

Figure S1: Funnel plot of effect sizes.

Table S1: Raw data for meta-analysis.

Data ID	Article	Species	Sex	Start Age (day)	Test n	Test Meas	Test SEM	Ctrl n	Ctrl Meas	Ctrl SEM	Units	Trait	Method	TimePt (days)	Fast time (hrs)	Anaes G/I (Y/N)	Fat (% E)	Test ID	Ctrl ID	Note/Flag
3	R14	Wistar	F		14	315.888	1.869	14	327.103	5.607	g	BW	Gravimetric	63		No	16	t.02	c.02	Average wks 8-10. Using midpoint of 63 days
5	R14	Wistar	F		14	5.9	0.3	14	5.9	0.2	mmol/L	Fast Glu	Basal IPGTT	70	15	No	16	t.02	c.02	overnight fast; no anaesthesia mentioned for IPGTT
4	R14	Wistar	F		14	1523	72	14	1322	55	mM.min	Glu AUC	IPGTT	70	15	No	16	t.02	c.02	End of lactation; overnight fast; no anaesthesia mentioned for IPGTT
6	R14	Wistar	F		14	5.68	0.64	14	5.67	0.89	% BW	BF	necropsy	70		No	16	t.02	c.02	
170	R14	Wistar	F		14	873.3214	47.85354	14	927.1554	47.85354	kJ/kg/day	E intake	by food int.	70		No	16	t.02	c.02	During lactation; Weeks 8-10
18	M09	C57BL/6J	F	49	7	17.9	0.8	7	19.3	1.7	%	BF	DEXA	154		No	13.5	t.07	c.07	Low fat diet only; Predicted GIs 53 and 102. Start age 6-8 wk. Used average of 49 days.
19	M09	C57BL/6J	F	49	7	7.7	0.7	7	5.7	0.5	mM	Fast Glu	NS	154	12	Yes	13.5	t.07	c.07	
20	M09	C57BL/6J	F	49	7	136	21	7	104	9	pM	Fast Ins	NS	154	12	Yes	13.5	t.07	c.07	
21	M09	C57BL/6J	F	49	7	26.9203	0.57975	7	26.34055	0.7971	g	BW	Gravimetric	154		No	13.5	t.07	c.07	
171	M09	C57BL/6J	F	49	7	46.2	1.1	7	42.3	1.2	kJ/day	E intake	by food int.	154		No	13.5	t.07	c.07	Mean weeks 0-22
22	R10	SD	M	126	11	46.4	1.98	10	33.2	2.53	g	BF	necropsy	28		No	16.7	t.08	c.08	AL; Ad lib (AL) and energy rest (ER); 4 wks HF to establish DIO rats; Assume Adult = 18wks
23	R10	SD	M	126	12	23.2	1.72	12	26.7	2.76	g	BF	necropsy	28		No	16.7	t.09	c.09	ER
24	R10	SD	M	126	11	6.8	0.19	10	6.1	0.27	mmol/L	Fast Glu	necropsy	28	12	No	16.7	t.08	c.08	AL
25	R10	SD	M	126	12	6.9	0.2	12	6.8	0.34	mmol/L	Fast Glu	necropsy	28	12	No	16.7	t.09	c.09	ER
26	R10	SD	M	126	11	417	27.3	10	246.3	15.1	pmol/L	Fast Ins	necropsy	28	12	No	16.7	t.08	c.08	AL
27	R10	SD	M	126	12	270.1	22.6	12	278.7	24.8	pmol/L	Fast Ins	necropsy	28	12	No	16.7	t.09	c.09	ER
28	R10	SD	M	126	11	591.784	10.798	10	568.31	12.4415	g	BW	Gravimetric	28		No	16.7	t.08	c.08	AL
29	R10	SD	M	126	12	583.803	7.042	12	503.052	5.8685	g	BW	Gravimetric	28		No	16.7	t.09	c.09	ER
30	R10	SD	M	126	5	1453	63.4	5	1294	46.1	mM/min	Glu AUC	OGTT	28	12	No	16.7	t.08	c.08	AL; sample size 5-8; no anaesthesia mentioned for OGTT
31	R10	SD	M	126	5	1202	18.3	5	1245	45.7	mM/min	Glu AUC	OGTT	28	12	No	16.7	t.09	c.09	ER; sample size 5-8
32	R10	SD	M	126	5	4.7	0.04	5	4.5	0.11	Log pM/min	Ins AUC	OGTT	28	12	No	16.7	t.08	c.08	AL; sample size 5-8
33	R10	SD	M	126	5	4.6	0.03	5	4.6	0.03	Log pM/min	Ins AUC	OGTT	28	12	No	16.7	t.09	c.09	ER; sample size 5-8
172	R10	SD	M	126	11	11	0.17	10	9.2	0.24	MJ/4 wk	E intake	by food int.	28		No	16.7	t.08	c.08	AL
173	R10	SD	M	126	12	6.9	0.01	12	7	0.05	MJ/4 wk	E intake	by food int.	28		No	16.7	t.09	c.09	ER
34	R11	SD	M	63	16	485	15	16	461	14	g	BW	DXA	28		No	16.7	t.10	c.10	0% vs 16%; Obesity Prone (OP) and res (OR); The n is always half each O phenotype; 5 diets: 0, 4, 8, 12 and 16% RS
35	R11	SD	M	63	16	465	12	16	461	14	g	BW	DXA	28		No	16.7	t.11	c.10	4% vs 16%
36	R11	SD	M	63	16	445	18	16	461	14	g	BW	DXA	28		No	16.7	t.12	c.10	8% vs 16%
37	R11	SD	M	63	16	474	11	16	461	14	g	BW	DXA	28		No	16.7	t.13	c.10	12% vs 16%
38	R11	SD	M	63	16	0.608696	0.478264	16	-5.15217	0.52174	%	BF	DXA	28		No	16.7	t.10	c.10	0% vs 16%
39	R11	SD	M	63	16	0.804348	0.478262	16	-5.15217	0.52174	%	BF	DXA	28		No	16.7	t.11	c.10	4% vs 16%
40	R11	SD	M	63	16	-1.23913	0.47826	16	-5.15217	0.52174	%	BF	DXA	28		No	16.7	t.12	c.10	8% vs 16%
41	R11	SD	M	63	16	-3.26087	0.5	16	-5.15217	0.52174	%	BF	DXA	28		No	16.7	t.13	c.10	12% vs 16%
42	R11	SD	M	63	8	6.4	0.2	8	6.3	0.2	mmol/L	Fast Glu	tail vein	21	12	Yes	16.7	t.10	c.10	0% vs 16%
43	R11	SD	M	63	8	5.9	0.2	8	6.3	0.2	mmol/L	Fast Glu	tail vein	21	12	Yes	16.7	t.11	c.10	4% vs 16%
44	R11	SD	M	63	8	6	0.2	8	6.3	0.2	mmol/L	Fast Glu	tail vein	21	12	Yes	16.7	t.12	c.10	8% vs 16%
45	R11	SD	M	63	8	6.3	0.2	8	6.3	0.2	mmol/L	Fast Glu	tail vein	21	12	Yes	16.7	t.13	c.10	12% vs 16%
46	R11	SD	M	63	8	241	52	8	293	52	pmol/L	Fast Ins	tail vein	21	12	Yes	16.7	t.10	c.10	0% vs 16%
47	R11	SD	M	63	8	275	52	8	293	52	pmol/L	Fast Ins	tail vein	21	12	Yes	16.7	t.11	c.10	4% vs 16%
48	R11	SD	M	63	8	172	52	8	293	52	pmol/L	Fast Ins	tail vein	21	12	Yes	16.7	t.12	c.10	8% vs 16%
49	R11	SD	M	63	8	224	52	8	293	52	pmol/L	Fast Ins	tail vein	21	12	Yes	16.7	t.13	c.10	12% vs 16%
174	R11	SD	M	63	16	378	12	16	338	10	kJ/day	E intake	by food int.	14		No	16.7	t.10	c.10	0% vs 16%
175	R11	SD	M	63	16	364	7	16	338	10	kJ/day	E intake	by food int.	14		No	16.7	t.11	c.10	4% vs 16%
176	R11	SD	M	63	16	355	12	16	338	10	kJ/day	E intake	by food int.	14		No	16.7	t.12	c.10	8% vs 16%
177	R11	SD	M	63	16	345	12	16	338	10	kJ/day	E intake	by food int.	14		No	16.7	t.13	c.10	12% vs 16%
50	R12	Wistar	M	49	6	268	11.7	6	246	10.7	g	BW	Gravimetric	56		No	2.5	t.14	c.11	Only high digestible starch (HDSD) and high resistant

																				starch (HRSD)
51	R12	Wistar	M	49	6	98	4	6	121	3	mg/dL	Fast Glu	necropsy	56	12	Yes	2.5	t.14	c.11	
52	R12	Wistar	M	49	6	1.3	0.3	6	0.9	0.2	ng/dL	Fast Ins	necropsy	56	12	Yes	2.5	t.14	c.11	
53	R12	Wistar	M	49	6	13404.3	851	6	13191.5	638.3	mg/dL.min	Glu AUC	OGTT	54	12	No	2.5	t.14	c.11	no anaesthesia mentioned for OGTT
178	R12	Wistar	M	49	6	628	8.7	6	845	44.4	average Cal	E intake	by food int.	56		No	2.5	t.14	c.11	
54	R01	SD	M	56	6	432.178	6.9305	6	422.277	4.4555	g	BW	Gravimetric	63		No	11	t.15	c.12	Every rat group had surgery to fit indwelling cannula 1 wk before their respective IVGTT
55	R01	SD	M	56	4	863	215	3	237	75	pM/120min	Ins AUC	IVGTT	63	15	No	11	t.15	c.12	Overnight fast stated; no anaesthesia mentioned for IVGTT
56	R01	Wistar	M	24	24	333.5	6.75	24	312.5	4.5	g	BW	Gravimetric	84		No	11	t.16	c.13	Start age 3-4 weeks; using midpoint 24 days
57	R01	Wistar	M	24	5	699	52	5	326	35	nM/120 min	Ins AUC	IVGTT	84	15	No	11	t.16	c.13	Overnight fast stated; no anaesthesia mentioned for IVGTT
58	R01	Wistar	M	24	5	1.7	0.3	5	0.9	0.1	pmol/L	Fast Ins	Basal IVGTT	84	15	No	11	t.16	c.13	Overnight fast stated; no anaesthesia mentioned for IVGTT
60	M10	C57BL/6	M	49	8	33.0814	1.30815	7	31.8605	1.56975	g	BW	Gravimetric	112		No	15	t.17	c.14	LF diets only; start age 6-8 weeks - using midpoint 49 days
61	M10	C57BL/6	M	49	8	151	8.6	7	149	6.3	mg/dL	Fast Glu	retro orbital plexus	112	5.5	Yes	15	t.17	c.14	Fast 5-6 hours; At death
62	M10	C57BL/6	M	49	8	0.39	0.04	7	0.37	0.01	ng/dL	Fast Ins	retro orbital plexus	112	5.5	Yes	15	t.17	c.14	Fast 5-6 hours; At death
180	M10	C57BL/6	M	49	8	47.6289	3.3677	7	53.6426	4.811	kJ/24h/mouse	E intake	by food int.	56		No	15	t.17	c.14	Average week 4 and 12 - using midpoint of week 8
63	R02	Wistar	M	42	36	435.738	5.453	36	412.248	4.614	g	BW	Gravimetric	112		No	11	t.18+t.19	c.15+c.16	HGI vs LGI; All groups cannulated 4 days prior to respective IVGTTs
64	R02	Wistar	M	42	6	364.43	14.681	36	412.248	4.614	g	BW	Gravimetric	112		No	11	t.20	c.15+c.16	GLUcose vs LGI
65	R02	Wistar	M	42	5	39.3773	4.7619	5	31.8681	6.5934	nM.30min	Ins AUC	IVGTT	364	20	No	11	t.18	c.15	HGI vs LGI; sample size 5-6; Fast overnight but Basal(/Fast Ins) stated 20hours; no anaesthesia mentioned for IVGTT
66	R02	Wistar	M	42	6	37.6	3.2	5	14.8993	3.0873	nM.30min	Ins AUC	IVGTT	112	20	No	11	t.19	c.16	HGI vs LGI; Fast overnight but Basal(/Fast Ins) stated 20hours; no anaesthesia mentioned for IVGTT
67	R02	Wistar	M	42	5	33.8255	2.4161	5	14.8993	3.0873	nM.30min	Ins AUC	IVGTT	112	20	No	11	t.20	c.16	GLU vs LGI; Fast overnight but Basal(/Fast Ins) stated 20hours; no anaesthesia mentioned for IVGTT
69	R02	Wistar	M	42	5	330.882	47.794	5	73.5294	18.3824	pmol/L	Fast Ins	Basal IVGTT	112	20	No	11	t.20	c.15+c.16	GLU vs LGI; no anaesthesia mentioned for IVGTT
70	R02	Wistar	M	42	5	827.206	172.794	5	610.294	84.559	pmol/L	Fast Ins	Basal IVGTT	364	20	No	11	t.18	c.15	HGI vs LGI; no anaesthesia mentioned for IVGTT
71	M11	C57BL/6J	M	112	10	32.8671	0.97905	10	30	0.4895	g	BW	Gravimetric	140		No	12	t.21	c.17	
72	M11	C57BL/6J	M	112	10	6.14054	0.69189	10	3.89189	0.17297	g	BF	NMR	112		No	12	t.21	c.17	
73	M11	C57BL/6J	M	112	10	6350	364	10	3714	548	mM/min	Glu AUC	IPGTT	105	15	No	12	t.21	c.17	Overnight fast stated; no anaesthesia mentioned for IPGTT
74	M11	C57BL/6J	M	112	10	14382	1541	10	13597	1054	pM/min	Ins AUC	IPGTT	105	15	No	12	t.21	c.17	Overnight fast stated; no anaesthesia mentioned for IPGTT
75	M11	C57BL/6J	M	112	10	14.3784	0.7568	10	12.2973	1.1351	mmol/L	Fast Glu	Basal IPGTT	105	15	No	12	t.21	c.17	Overnight fast stated; no anaesthesia mentioned for IPGTT
76	M11	C57BL/6J	M	112	10	78.4946	12.043	10	51.828	17.2043	pmol/L	Fast Ins	Basal IPGTT	105	15	No	12	t.21	c.17	Overnight fast stated; no anaesthesia mentioned for IPGTT
183	M11	C57BL/6J	M	112	10	5904.8	108.4	10	5610.1	99.4	kJ/18wk	E intake	by food int.	126		No	12	t.21	c.17	
77	M07	C57BL/6J	M	112	8	32.3026	1.08555	8	29.9342	0.5263	g	BW	Gravimetric	140		No	12	t.22	c.18	WT only; sample size 8-10 for all
78	M07	C57BL/6J	M	308	8	33.1579	0.8553	8	31.0526	0.4934	g	BW	Gravimetric	182		No	12	t.23	c.19	
79	M07	C57BL/6J	M	112	8	6.16471	0.65882	8	3.85882	0.18824	g	BF	NMR	112		No	12	t.22	c.18	
80	M07	C57BL/6J	M	308	8	6.58824	0.8	8	4.51765	0.75294	g	BF	NMR	168		No	12	t.23	c.19	
81	M07	C57BL/6J	M	112	8	6176.47	329.41	8	3952.94	494.12	mM*min	Glu AUC	IPGTT	105	15	No	12	t.22	c.18	Overnight fast stated; no anaesthesia mentioned for IPGTT
82	M07	C57BL/6J	M	308	8	3435.29	188.24	8	2870.59	376.47	mM*min	Glu AUC	IPGTT	140	15	No	12	t.23	c.19	Overnight fast stated; no anaesthesia mentioned for IPGTT
83	M07	C57BL/6J	M	112	8	14.02355	0.58925	8	12.2559	0.9428	mmol/L	Fast Glu	Basal IPGTT	105	15	No	12	t.22	c.18	Overnight fast stated; no anaesthesia mentioned for IPGTT
84	M07	C57BL/6J	M	308	8	10.325	1.399985	8	8.691665	0.64165	mmol/L	Fast Glu	Basal IPGTT	140	15	No	12	t.23	c.19	Overnight fast stated; no anaesthesia mentioned for IPGTT
186	M07	C57BL/6J	M	112	8	5882.35	117.65	8	5705.88	117.65	kJ/18 wk	E intake	NET: by food intake	126		No	12	t.24	c.20	
189	M07	C57BL/6J	M	308	8	7588.24	117.64	8	7000	176.47	kJ/22 wk	E intake	NET: by food intake	154		No	12	t.25	c.21	
85	M17	C57BL/6	M	49	16	37.9729	1.28135	17	34.4949	0.5797	g	BW	Gravimetric	105		No	13.4	t.24	c.20	First 16 wks only; start age 6-8 weeks, using midpoint 49 days
86	M17	C57BL/6	M	49	15	29.7	7	17	23.2	0.6	%	BF	EchoMRI	105		No	13.4	t.24	c.20	
87	M17	C57BL/6	M	49	15	92	5.163978	15	94	3.614784	mg/dL	Fast Glu	Basal OGTT	98	12	No	13.4	t.24	c.20	Sample size 15-17; no anaesthesia mentioned for OGTT
88	M17	C57BL/6	M	49	15	6850	339.5315	15	6192	529.3077	min*mg/dL	Glu AUC	OGTT	98	12	No	13.4	t.24	c.20	Sample size 15-17; no anaesthesia mentioned for OGTT
191	M17	C57BL/6	M	49	15	0.612056	0.050375	16	0.707769	0.052894	kcal/g bw/day	E intake	f int.*density	105		No	13.4	t.24	c.20	
89	R03	SD	M	42	14	376	9	14	355	4	g	BW	Gravimetric	35		No	17.5	t.25	c.21	
90	R03	SD	M	42	14	326	18	14	316	8	g	BW	Gravimetric	35		No	17.5	t.26	c.22	STZ; STZ = neonatal streptozotocin

91	R06	Wistar	M	45	10	336	11	10	327	11	g	BW	Gravimetric	49		No	11	t.27	c.23	Has HF morning meal so daily food 45% of E Carb; surgery to insert cannula 6-7 days prior to clamp; Start age 6-7 weeks, using midpoint 45 days
92	R06	Wistar	M	45	10	315	30	10	253	32	mmol/(L.60min)	Glu AUC	IVGTT	63	15	No	11	t.27	c.23	Switched numbers based on graph; Stated food-deprived; no anaesthesia mentioned for IVGTT
93	R06	Wistar	M	45	10	36610	3913.1	10	25360	3478.3	pmol/(L.30min)	Ins AUC	IVGTT	63	15	No	11	t.27	c.23	Stated food-deprived; no anaesthesia mentioned for IVGTT
94	R06	Wistar	M	45	7	5.26	0.06	7	5.31	0.07	mmol/L	Fast Glu	clamp	49	15	No	11	t.27	c.23	Stated food-deprived; no anaesthesia mentioned for clamp
95	R06	Wistar	M	45	7	232	32	7	210	14	pmol/L	Fast Ins	clamp	49	15	No	11	t.27	c.23	Stated food-deprived; no anaesthesia mentioned for clamp
96	M02	C57BL/6J	M	77	10	31.79	0.83	10	30.21	0.49	g	BW	Gravimetric	63		No	11	t.28	c.24	
97	M02	C57BL/6J	M	77	10	8.13	0.81	10	4.21	0.67	g	BF	tritiated water injection	63		No	11	t.28	c.24	
99	R07	SD	M	42	11	547.9	13.4	10	549.2	15.2	g	BW	Gravimetric	119		No	11	t.29	c.25	Rats partially pancreatectomised 2 wks prior to start (wk -2: 8 wk age)
100	R07	SD	M	42	11	97.8	13.6	10	57.3	7.2	g	BF	tritiated water injection	119		No	11	t.29	c.25	
102	R07	SD	M	42	11	65.4	37	10	-85.8	24.4	mM 120 min	Glu AUC	OGTT	98	16	No	11	t.29	c.25	versus day 0; no anaesthesia mentioned for OGTT
103	R07	SD	M	42	11	592	50.4	10	261.4	39.8	pM 120 min	Ins AUC	OGTT	98	16	No	11	t.29	c.25	versus day 0; no anaesthesia mentioned for OGTT
104	R07	SD	M	42	11	4.83871	0.19355	10	4.51613	0.12903	mmol/L	Fast Glu	Basal OGTT	98	16	No	11	t.29	c.25	no anaesthesia mentioned for OGTT
105	R07	SD	M	42	11	5.45161	1	10	2.935485	1	pmol/L	Fast Ins	Basal OGTT	98	16	No	11	t.29	c.25	no anaesthesia mentioned for OGTT
106	R07	SD	M	42	3.5	468.7	14.9	3.5	458.5	17.5	g	BW	Gravimetric	49		No	11	t.30	c.26	Partially pancreatomised at wk -2. Switched to opposite diet after 7 wks
107	R07	SD	M	42	3.5	515.7	20.6	3.5	500.6	18.9	g	BW	Gravimetric	70		No	11	c.26	t.30	
108	R07	SD	M	42	7	239.9	47.1	7	-85.9	51.1	mM 120 min	Glu AUC	OGTT	70	16	No	11	c.26 vs t.30	t.30 vs c.26	versus day 49; no anaesthesia mentioned for OGTT
109	R07	SD	M	42	7	522	58.2	7	-38.5	58.6	pM 120 min	Ins AUC	OGTT	70	16	No	11	c.26 vs t.30	t.30 vs c.26	versus day 49; no anaesthesia mentioned for OGTT
110	M05	129SvPas	M	35	5	35.2	3.264659	5	33.1	0.626099	g	BW	Gravimetric	266		No	13	t.31	c.27	Sample size 5-8
111	M05	129SvPas	M	35	5	30.1	3.398823	5	21.5	1.386362	%	BF	DEXA	266		No	13	t.31	c.27	Sample size 5-8
112	M05	129SvPas	M	35	4	6.2	0.25	4	5.4	0.45	mmol/L	Fast Glu	Basal OGTT	245	5.5	No	13	t.31	c.27	Sample size 4-8; Fast 5-6 hours; no anaesthesia mentioned for OGTT
113	M05	129SvPas	M	35	4	114.5	34	4	58.8	11.35	pmol/L	Fast Ins	Basal OGTT	245	5.5	No	13	t.31	c.27	Sample size 4-8; Fast 5-6 hours; no anaesthesia mentioned for OGTT
114	M05	129SvPas	M	35	4	2061.22	183.675	4	1897.96	122.45	total AUC	Glu AUC	OGTT	245	5.5	No	13	t.31	c.27	Sample size 4-8; Fast 5-6 hours; no anaesthesia mentioned for OGTT
115	M05	129SvPas	M	35	4	108	24	4	48	5.5	total AUC	Ins AUC	OGTT	245	5.5	No	13	t.31	c.27	Sample size 4-8; Fast 5-6 hours; no anaesthesia mentioned for OGTT
205	M05	129SvPas	M	35	7	20.3279	1.239232	7	20.3279	1.610998	kcal/48hr	E intake	NET: by food intake	266		No	13	t.31	c.27	Sample size 6-8
116	M03	129S2/SvPas	M	35	7	32.2449	0.925635	7	30.7483	0.925635	g	BW	Gravimetric	168		No	13	t.32	c.28	Sample size 7-9
117	M03	129S2/SvPas	M	35	6	12.2	1.18392	6	6.1	1.714643	%	BF	DXA	126		No	13	t.32	c.28	Sample size 6-9
118	M03	129S2/SvPas	M	35	3.5	98.41	3.76377	3.5	94.8954	4.870677	mg/dL	Fast Glu	tail vein	105	15	No	13	t.32	c.28	Sample size 7-8; no anaesthesia mentioned
119	M03	129S2/SvPas	M	35	3.5	59.749	1.549806	3.5	62.6778	1.771217	mg/dL	Fast Glu	tail vein	105	6	No	13	t.32	c.28	Sample size 7-8; no anaesthesia mentioned
120	M03	129S2/SvPas	M	35	7	0.513889	0.062994	7	0.263889	0.026247	ng/mL	Fast Ins	tail vein	105	6	No	13	t.32	c.28	Sample size 7-8; no anaesthesia mentioned
206	M03	129S2/SvPas	M	35	7	80.198	1.400213	7	84.1584	1.575257	kcal/wk	E intake	by food int.	168		No	13	t.32	c.28	Sample size 7-9
121	M04	C57BL/6	M	21	20	106.23	48.89	16	75.25	44.33	pmol/L	Fast Ins	necropsy	56	16	Yes	11	t.33	c.29	Cardiac puncture following 1H NMR(which used anaesthesia)
122	M04	C57BL/6	M	21	20	16.89	5.79	16	17.4	4.8	mmol/L	Fast Glu	necropsy	56	16	Yes	11	t.33	c.29	Cardiac puncture following 1H NMR(which used anaesthesia)
123	M04	C57BL/6	M	21	20	25.88865	1.48105	16	15.8769	2.48805	%	BF	1H MRS	56		No	11	t.33	c.29	
124	M04	C57BL/6	M	21	20	29.3368	0.4421	16	28.6737	0.6632	g	BW	Gravimetric	56		No	11	t.33	c.29	
214	M04	C57BL/6	M	21	20	345.61	5.122	16	298.4145	4.0245	kJ/mouse/wk	E intake	by food int.	56		No	11	t.33	c.29	
128	M08	C57BL/6JOlaHsd	M	63	50	32.98	0.5	50	31.2	0.4	g	BW	Gravimetric	91		No	30	t.35+t.36+t.37	c.31+c.32+c.33	
129	M08	C57BL/6JOlaHsd	M	63	40	685.714	44.721	40	496.894	39.752	pg/mL	Fast Ins	necropsy	91	2	Yes	30	t.35+t.36+t.37	c.31+c.32+c.33	
130	M08	C57BL/6JOlaHsd	M	63	40	9.375	0.1875	40	9	0.28125	mM	Fast Glu	necropsy	91	2	Yes	30	t.35+t.36+t.37	c.31+c.32+c.33	
134	M08	C57BL/6JOlaHsd	M	63	7	701.22	24.39	7	548.78	18.2925	glucose*time; au	Glu AUC	IPGTT	77	5	No	30	t.37	c.33	Sample size 7-10; no anaesthesia mentioned for IPGTT
215	M08	C57BL/6JOlaHsd	M	63	50	1350	20	50	1355	20	kcal/13 wk	E intake	by food int.	91		No	30	t.37	c.33	
136	M01	CBA/T6	M	42	12	31.1	0.6	12	29	0.4	g	BW	Gravimetric	70		No	11	t.39	c.35	WT; GTG = IP injection of gold thioglucose at Week 2
137	M01	CBA/T6	M	42	12	38.1	0.6	11	35.6	0.8	g	BW	Gravimetric	70		No	11	t.40	c.36	GTG

138	M01	CBA/T6	M	42	12	1.93	0.29	12	2.82	0.37	ng/mL	Fast Ins	necropsy	70	2.5	Yes	11	t.39	c.35	WT; Fast 2-3 hours
139	M01	CBA/T6	M	42	12	3.83	0.61	11	5.34	0.55	ng/mL	Fast Ins	necropsy	70	2.5	Yes	11	t.40	c.36	GTG; Fast 2-3 hours
140	M01	CBA/T6	M	42	12	13.6	0.7	12	12.2	0.6	mM	Fast Glu	necropsy	70	2.5	Yes	11	t.39	c.35	WT; Fast 2-3 hours
141	M01	CBA/T6	M	42	12	18	1.4	11	14.3	0.5	mM	Fast Glu	necropsy	70	2.5	Yes	11	t.40	c.36	GTG; Fast 2-3 hours
142	R05	Wistar	M	42	23	340	4	26	305	3	g	BW	Gravimetric	42		No	11	t.41+t.42	c.37+c.38	
143	R05	Wistar	M	42	8	5.4	0.3	8	5.7	0.3	mmol/L	Fast Glu	Basal IVGTT	42	15	No	11	t.41	c.37	Control; Lep rats given bolus recombinant mouse leptin followed by continuous infusion for 1 hr. Control rats received vehicle only; rats had cannula surgery 3-4 days prior IVGTT
144	R05	Wistar	M	42	5	5.9	0.2	6	5.7	0.1	mmol/L	Fast Glu	Basal IVGTT	42	15	No	11	t.42	c.38	Lep; overnight fast; no anaesthesia mentioned for IVGTT
145	R05	Wistar	M	42	8	2	0.2	8	1.6	0.3	ng/mL	Fast Ins	Basal IVGTT	42	15	No	11	t.41	c.37	Control; overnight fast; no anaesthesia mentioned for IVGTT
146	R05	Wistar	M	42	5	1.7	0.3	6	0.9	0.2	ng/mL	Fast Ins	Basal IVGTT	42	15	No	11	t.42	c.38	Lep; overnight fast; no anaesthesia mentioned for IVGTT
147	R05	Wistar	M	42	8	590	26	8	587	20	mM x h	Glu AUC	IVGTT	42	15	No	11	t.41	c.37	Control; overnight fast; no anaesthesia mentioned for IVGTT
148	R05	Wistar	M	42	5	633	12	6	653	11	mM x h	Glu AUC	IVGTT	42	15	No	11	t.42	c.38	Lep; overnight fast; no anaesthesia mentioned for IVGTT
149	R05	Wistar	M	42	8	707	163	8	200	30	ng/mL x h	Ins AUC	IVGTT	42	15	No	11	t.41	c.37	Control; overnight fast; no anaesthesia mentioned for IVGTT
150	R05	Wistar	M	42	5	298	48	6	214	26	ng/mL x h	Ins AUC	IVGTT	42	15	No	11	t.42	c.38	Lep; overnight fast; no anaesthesia mentioned for IVGTT
151	M06	C57BL/6J	M	126	8	156.0605	7.5755	8	190.909	18.182	mg/dL	Fast Glu	Basal OGTT	19	5	No	16.7	t.43	c.39	Ctrl HGI vs STZ LGI; STZ = streptozotocin injection once daily for Days 11-15. Control mice received a citrate buffer injection; Assume Adult = 18 wks; Sample size 8-9; no anaesthesia mentioned for OGTT
152	M06	C57BL/6J	M	126	8	251.515	24.2425	8	190.909	18.182	mg/dL	Fast Glu	Basal OGTT	19	5	No	16.7	t.44	c.39	STZ HGI vs STZ LGI; Sample size 8-9; no anaesthesia mentioned for OGTT
153	M06	C57BL/6J	M	126	8	17.0504	0.8633	8	20.9353	1.5107	g/dL*180min	Glu AUC	OGTT	19	5	No	16.7	t.43	c.39	Ctrl HGI vs STZ LGI; Sample size 8-9; no anaesthesia mentioned for OGTT
154	M06	C57BL/6J	M	126	8	26.7626	1.7266	8	20.9353	1.5107	g/dL*180min	Glu AUC	OGTT	19	5	No	16.7	t.44	c.39	STZ HGI vs STZ LGI; Sample size 8-9; no anaesthesia mentioned for OGTT
155	R09	SD	M	126	50	4.39	0.1	50	3.55	0.11	g	BF	necropsy	10		No	16.7	t.45	c.40	Can't tell of F1 is fed or fasted Glu/Ins; Assume Adult = 18 wks
216	R09	SD	M	126	50	133.65	1.65	50	130.35	2.97	kcal/48 hour	E intake	by food int.	9		No	16.7	t.45	c.40	
157	R04	SD	F		14	275	3	16	262	6	g	BW	Gravimetric	14		No	11	t.46	c.41	Sham result; glucose = HGI, vs fructose = LGI; Start age not stated
158	R04	SD	F		14	342	6	12	330	14	g	BW	Gravimetric	14		No	11	t.47	c.42	VMH results
159	R04	SD	F		14	1.5	0.3	16	2.1	0.3	ng/mL	Fast Ins		14	5	Yes	11	t.46	c.41	Sham result; no anaesthesia mentioned for IVGTT but is mentioned for clamp
160	R04	SD	F		14	3.2	0.5	12	3.5	0.3	ng/mL	Fast Ins		14	5	Yes	11	t.47	c.42	VMH results; no anaesthesia mentioned for IVGTT but is mentioned for clamp
161	R04	SD	F		14	6.9	0.4	16	7.3	0.4	mmol/L	Fast Glu		14	5	Yes	11	t.46	c.41	Sham result; no anaesthesia mentioned for IVGTT but is mentioned for clamp
162	R04	SD	F		14	4.3	0.3	12	6.6	0.5	mmol/L	Fast Glu		14	5	Yes	11	t.47	c.42	VMH results; no anaesthesia mentioned for IVGTT but is mentioned for clamp
163	R04	SD	F		6	353.623	104.348	6	823.188	168.116	ng/mL*min	Ins AUC	IVGTT	14	5	No	11	t.46	c.41	Sham result; no anaesthesia mentioned for IVGTT
164	R04	SD	F		5	405.797	63.768	5	736.232	226.087	ng/mL*min	Ins AUC	IVGTT	14	5	No	11	t.47	c.42	VMH results; no anaesthesia mentioned for IVGTT
165	M16	C57Bl/6J	M	77	9	1921.82	97.72	10	1425.08	73.29	mM.min	Glu AUC	IPGTT	231	6	No	14	t.48	c.43	WT only; no anaesthesia mentioned for IPGTT; units not stated; assuming IPGTT occurred at end
166	M16	C57Bl/6J	M	77	9	11.3055	0.4244	10	8.8746	0.34726	mmol/L	Fast Glu	Average of 4	231	6	No	14	t.48	c.43	ASSUMPTIONS based on limited data
167	M16	C57Bl/6J	M	77	9	30.0645	2.4516	10	28.3871	1.6774	g	BW	Gravimetric	231		No	14	t.48	c.43	

Article number refers to list in Tables 4/6 and 5/7 for mice and rats respectively. Articles M12 (Van Schothorst *et al.* 2011), M13 (Uchiki *et al.* 2012), M14 (Weikel *et al.* 2012), M15 (Birarda *et al.* 2013), R08 (Kopilas *et al.* 2007), R13 (Stavrovskaya *et al*, 2013) and R15 (Thompson *et al.* 2016) did not contain any relevant results. Test/Ctrl ID refers to the cohort used. Data ID is unique.

Text S1: R code for meta-analysis.

```
rm(list=ls())
detach(data)


# install.packages("metafor")

library(metafor)

setwd("X:")

data<-read.csv("HGIdata1.csv")


# Changing SEM to SD for S pooled calculation

data$Ctrl.SD<-data$Ctrl.SEM*sqrt(data$Ctrl.n)

data$Test.SD<-data$Test.SEM*sqrt(data$Test.n)


#Scaling continuous variables to units of SD instead of unit (day/hour/%)

data$Start.Age..day.<-scale(data$Start.Age..day.)

data$TimePt..days.<-scale(data$TimePt..days.)

data$Fast.time..hrs.<-scale(data$Fast.time..hrs.)

data$Fat....E.<-scale(data$Fat....E.)


# Step 1: Calculate effect sizes

J<-1-(3/(4*(data$Ctrl.n+data$Test.n-2)-1))

spooled<-sqrt((((data$Ctrl.n-1)*(data$Ctrl.SD)^2+(data$Test.n1)*(data$Test.SD)^2)/(data$Ctrl.n+data$Test.n-2))

data$d<-(data$Test.Meas-data$Ctrl.Meas)/spooled*J

data$variance<-(data$Ctrl.n+data$Test.n)/(data$Ctrl.n*data$Test.n)+(data$d^2/(2*(data$Test.n+data$Ctrl.n)))


Calc.cov.d<-function(CN, d_i, d_j, Total.N){

  Cov<-(1 / CN) + ((d_i * d_j) / (2 * Total.N))

  return(Cov)

}


#create vector of all 6 unique traits to loop through for each

treatments<-unique(data$Trait)


panels<-c("a","a","c","b","c","b","d")
```

```
title<-c("Body Weight","Fasting Glucose","Glucose AUC","Body Fat","Energy Intake","Fasting Insulin","Insulin AUC")
pdf("Figures 2 and 3.pdf", 11.69, 8.27)
par(mfrow=c(3,5), mar=c(5,1,3,1))
plot(-10, -10, ylim=c(0,11), xlim=c(-2,2),xlab="",ylab="",axes=F)

#pdf("Figure S1.pdf", 8.27, 11.69)
#par(mfrow=c(4,2), mar=c(5,5,3,1))

#loop
for(i in 1:length(treatments)){
 # get the appropriate subset of the data
 subset<-treatments[i]

 # Step 2: subset dataset example data.BW<-data[which(data$Trait == "BW"),]
 data.subset<-data[which(data$Trait == subset),]
 print(dim(data.subset))

 #creating varibale to enable dissection by mouse or rat
 MR<-substr(data.subset$Article, 1, 1)

 # Create matrix for VCV
 VCV.lnRR<-matrix(0,nrow = dim(data.subset)[1],ncol = dim(data.subset)[1])

 # get data IDs
 Data.ID<-data.subset$Data.ID
 rownames(VCV.lnRR) <- Data.ID
 colnames(VCV.lnRR) <- Data.ID

 # Find out those with shared controls
 Shared.Control<-data.subset$Ctrl.ID
 shared_coord <- which(Shared.Control%in%Shared.Control[duplicated(Shared.Control)]==TRUE)

 # If there are shared coords add in the covariance
```

```
if(length(shared_coord) > 0){

  # matrix of combinations of coordinates for each experiment with shared control
  combinations<-do.call("rbind",tapply(shared_coord, data.subset[shared_coord,"Ctrl.ID"], function(x) t(combn(x,2))))

  # Calculate covariance values between d values at the positions in shared_list and place them on the matrix
  for (j in 1:dim(combinations)[1]){
    p1 <- combinations[j,1]
    p2 <- combinations[j,2]
    p1_p2_cov <- Calc.cov.d(CN = data.subset[p1,"Ctrl.n"], d_i = data.subset[p1,"d"], d_j = data.subset[p2,"d"], Total.N
= (data.subset[p1,"Ctrl.n"] + data.subset[p1,"Test.n"] + data.subset[p2,"Test.n"]))
    VCV.lnRR[p1,p2] <- p1_p2_cov
    VCV.lnRR[p2,p1] <- p1_p2_cov
  }
}

# add in the diagonal as the Variances
diag(VCV.lnRR)<-data.subset$variance

# Using matrix to conduct more complex meta-analyses
Metac<-rma.mv(yi=d,V=VCV.lnRR,random=~Data.ID,data=data.subset)
summary(Metac)
resid<-rstandard(Metac)$resid
modeltf<-rma(yi=resid, vi=data.subset$variance)
trimfill(modeltf)
#funnel(Metac)
#mtext(title[i],side=3,line=1)

Metac.reg1<-rma.mv(yi=d,V=VCV.lnRR,random=~Data.ID,data=data.subset, mods=~TimePt..days.)
summary(Metac.reg1)

Metac.reg2<-rma.mv(yi=d,V=VCV.lnRR,random=~Data.ID,data=data.subset, mods=~Sex-1)
summary(Metac.reg2)
```

```
Metac.reg3<-rma.mv(yi=d,V=VCV.lnRR,random=~Data.ID,data=data.subset, mods=~Start.Age..day.)
summary(Metac.reg3)

#Metac.reg4<-rma.mv(yi=d,V=VCV.lnRR,random=~Data.ID,data=data.subset, mods=~Species)
#summary(Metac.reg4)

Metac.reg7<-rma.mv(yi=d,V=VCV.lnRR,random=~Data.ID,data=data.subset, mods=~MR-1)
summary(Metac.reg7)

Metac.reg8<-rma.mv(yi=d,V=VCV.lnRR,random=~Data.ID,data=data.subset, mods=~Fat....E.)
summary(Metac.reg8)

plot(-10, -10, ylim=c(0,11), xlim=c(-2,2),xlab="Effect Size",ylab="",axes=F)
axis(1)
abline(v=0)

points(Metac$b,11,pch=16)
arrows(Metac$ci.lb,11,Metac$ci.ub,11,code=0)
points(Metac.reg7$b,c(10,9),pch=16)
arrows(Metac.reg7$ci.lb, c(10,9),Metac.reg7$ci.ub,c(10,9), code=0)
points(Metac.reg3$b[2],4,pch=16)
arrows(Metac.reg3$ci.lb[2],4,Metac.reg3$ci.ub[2],4, code=0)
points(Metac.reg2$b,c(8,7),pch=16)
arrows(Metac.reg2$ci.lb, c(8,7),Metac.reg2$ci.ub,c(8,7), code=0)
points(Metac.reg1$b[2],6,pch=16)
arrows(Metac.reg1$ci.lb[2],6,Metac.reg1$ci.ub[2],6, code=0)
points(Metac.reg8$b[2],5,pch=16)
arrows(Metac.reg8$ci.lb[2],5,Metac.reg8$ci.ub[2],5, code=0)

if (i %in% c(2,3,6,7)){
  Metac.reg6<-rma.mv(yi=d,V=VCV.lnRR,random=~Data.ID,data=data.subset, mods=~Fast.time..hrs.)
  summary(Metac.reg6)
```

```
    points(Metac.reg6$b[2],3,pch=16)

    arrows(Metac.reg6$ci.lb[2],3,Metac.reg6$ci.ub[2],3, code=0)

  }


  if (i %in% c(2,6)){

    Metac.reg5<-rma.mv(yi=d,V=VCV.lnRR,random=~Data.ID,data=data.subset, mods=~Anaes.G.I..Y.N.-1)

    summary(Metac.reg5)

    points(Metac.reg5$b,c(2,1),pch=16)

    arrows(Metac.reg5$ci.lb, c(2,1),Metac.reg5$ci.ub,c(2,1), code=0)

  }


  mtext(title[i],side=3,line=1)

  mtext(panels[i],side=3,line=1,at=-1.5)



  if ( i < 3) {

    mtext("All",side=2,at=11,line=1,las=2)

    mtext("Mice",side=2,at=10,line=1,las=2)

    mtext("Rats",side=2,at=9,line=1,las=2)

    mtext("Starting Age",side=2,at=4,line=1,las=2)

    mtext("Female",side=2,at=8,line=1,las=2)

    mtext("Male",side=2,at=7,line=1,las=2)

    mtext("Length of Diet",side=2,at=6,line=1,las=2)

    mtext("Dietary Fat (% E)",side=2,at=5,line=1,las=2)

    mtext("Fasting Time",side=2,at=3,line=1,las=2)

    mtext("No Anaesthesia",side=2,at=2,line=1,las=2)

    mtext("Anaesthesia",side=2,at=1,line=1,las=2)

    }

}


dev.off()
```
